# C/EBPβ-Thr217 Phosphorylation Signaling Contributes to the Development of Lung Injury and Fibrosis in Mice

**DOI:** 10.1371/journal.pone.0025497

**Published:** 2011-10-05

**Authors:** Martina Buck, Mario Chojkier

**Affiliations:** 1 Department of Medicine, VA Healthcare Center, San Diego, California, United States of America; 2 Department of Medicine and Biomedical Sciences Program, University of California San Diego, La Jolla, California, United States of America; Erlangen University, Germany

## Abstract

**Background:**

Although C/EBPβ^ko^ mice are refractory to Bleomycin-induced lung fibrosis the molecular mechanisms remain unknown. Here we show that blocking the ribosomal S-6 kinase (RSK) phosphorylation of the CCAAT/Enhancer Binding Protein (C/EBP)-β on Thr217 (a RSK phosphoacceptor) with either a single point mutation (Ala217), dominant negative transgene or a blocking peptide containing the mutated phosphoacceptor ameliorates the progression of lung injury and fibrosis induced by Bleomycin in mice.

**Methodology/Principal Findings:**

Mice expressing the non-phosphorylatable C/EBPβ-Ala217 transgene had a marked reduction in lung injury on day-13 after Bleomycin exposure, compared to C/EBPβ^wt^ mice, judging by the decrease of CD68^+^ activated monocytes/macrophages, bone marrow-derived CD45^+^ cells and lung cytokines as well as by the normal surfactant protein-C expression by lung pneumocytes. On day-21 after Bleomycin treatment, C/EBPβ^wt^ mice but not mice expressing the dominant negative C/EBPβ-Ala217 transgene developed severe lung fibrosis as determined by quantitative collagen assays. All mice were of identical genetic background and back-crossed to the parental wild-type inbreed FVB mice for at least ten generations. Treatment of C/EBPβ^wt^ mice with a cell permeant, C/EBPβ peptide that inhibits phosphorylation of C/EBPβ on Thr217 (40 µg instilled intracheally on day-2 and day-6 after the single Bleomycin dose) also blocked the progression of lung injury and fibrosis induced by Bleomycin. Phosphorylation of human C/EBPβ on Thr266 (human homologue phosphoacceptor) was induced in collagen-activated human lung fibroblasts in culture as well as in activated lung fibroblasts in situ in lungs of patients with severe lung fibrosis but not in control lungs, suggesting that this signaling pathway may be also relevant in human lung injury and fibrosis.

**Conclusions/Significance:**

These data suggest that the RSK-C/EBPβ phosphorylation pathway may contribute to the development of lung injury and fibrosis.

## Introduction

The morbidity and mortality, as well as the financial and personal burden of lung injury and fibrosis are substantial. Lung fibrosis and the resulting abnormal pulmonary function are responsible for the associated complications, including pulmonary hypertension, cardiac failure, lung tumors, and cachexia [1]. All these complications of lung fibrosis are the main indication for lung transplantations in the US and worldwide [2]. According to the National Heart, Lung and Blood Institute, idiopathic pulmonary fibrosis (IPF) affects 5 million people worldwide and 200,000 patients in the US. No therapy is known to improve health-related quality of life or survival in patients with IPF [3] and these patients live only 3 to 5 years after diagnosis [4]. Therefore, preventing the progression, or inducing regression of lung injury and fibrosis will have a major potential impact in these patients.

Quiescent lung fibroblast produce negligible amounts of extracellular matrix proteins (ECM), but after their activation, these cells develop a myofibroblast phenotype, proliferate and become the main contributors of ECM [5]–[7]. This step is important for the development of lung fibrosis [1],[6],[7].

As reported by us and others, C/EBPβ^ko^ mice are refractory to hepatotoxin-induced liver fibrosis [8] and Bleomycin-induced lung fibrosis [9]. Although knock-out mice are a valuable initial tool to determine the general role of gene products, as in the case of C/EBPβ^ko^ mice being refractory to Bleomycin-induced lung fibrosis, knockout mice do not allow identification of the molecular mechanisms downstream from extracellular signaling cues. Knowledge of the specific signaling involving a single amino acid within a specific phosphoacceptor domain of the protein (in this case C/EBPβ) is necessary to understand the mechanisms and to eventually design effective targeted therapeutics that are still lacking in the treatment of human lung injury and fibrosis [1],[6],[7].

In this context, the mitogen-activated protein kinase (MAPK) pathway, through the extracellular signal-regulated kinase (ERK1/2), activates p-90 RSK [10]–[14], resulting in the phosphorylation of mouse C/EBPβ (NP_034013 XP_916631) on Thr217(Thr266 in human C/EBPβ) as we reported previously [10],[15]. The RSK phosphoacceptor site in C/EBPβ is identical in mouse and human, and it is evolutionary conserved [15].

We and others have established that site-specific phosphorylations of C/EBPβ are critical modulators of gene expression, cell-cycle progression, apoptotic programs, immune modulation and tissue inflammation and repair [8],[10],[15]–[17]. Our central hypothesis was that the RSK- C/EBPβ-Thr217 phosphorylation pathway may be critical for lung injury and fibrogenesis since as reported by us and others, this signaling is essential for both the macrophage inflammatory function [16],[17] and survival of activated liver myofibroblasts (hepatic stellate cells) [8],[10]. We proposed that macrophage inflammatory function and survival of activated lung myofibroblasts may determine the degree of lung injury and fibrosis.

Chronic Bleomycin treatment can induce lung injury and fibrosis in humans [18], and Bleomycin treatment is a classical method of inducing lung injury and fibrosis in mice [9],[18]–[21]. We used this model to investigate the role of RSK and phosphorylation of C/EBPβ on Thr217 in lung injury and fibrosis. Here we show that inhibition of C/EBPβ phosphorylation on Thr217 (RSK phosphoacceptor) within the transactivation domain with either a single point mutation (Ala217), dominant negative transgene or a blocking peptide containing the mutated phosphoacceptor ameliorates the progression of lung injury and fibrosis induced by Bleomycin in mice. Phosphorylation of human C/EBPβ on Thr266 (human homologue phosphoacceptor) was induced in collagen-activated human lung fibroblasts in culture as well as in activated lung fibroblasts in situ in lungs of patients with severe lung fibrosis but not in control lungs, suggesting that this signaling pathway may be also relevant in human lung injury and fibrosis.

## Results

### Mice expressing the dominant negative C/EBPβ-Ala217 transgene are resistant to Bleomycin-induced lung fibrosis

Given the important role of RSK and its phosphorylation of C/EBPβ-Thr217 in the activation of liver myofibroblasts (hepatic stellate cell) [10],[22] and the suggested similarities between liver and lung fibrogenesis [1],[23], we hypothesized that a dominant negative, non-phosphorylatable transgenic C/EBPβ-Ala217 would block phosphorylation of C/EBPβ-Thr217 by RSK, preventing lung myofibroblast (LMF) activation and decreasing lung fibrosis after lung injury.

Because the chronic exposure to the lung toxin Bleomycin can induce lung fibrosis in humans, and it is a classical method of inducing lung injury and fibrosis in mice [18],[24], we analyzed whether it induces lung fibrosis in mice expressing the dominant negative, nonphosphorylatable C/EBPβ-Ala217 transgene. This mutation changes the phosphorylatable Thr217 to a nonphosphorylatable Ala217 within the RSK phosphoacceptor site of C/EBPβ. These animals are developmentally normal, fertile and have a normal life span as we reported [8],[10] suggesting that the RSK-inhibitory transgene is apparently not toxic. The transgene is expressed to a similar degree that the C/EBPβ^wt^ in all tissues tested to date, including lungs. Specifically, the transgene is expressed in epithelial cells, macrophages and myofibroblasts. All the animals used in this study were of the same FVB genetic background with back-crossing for more than 10 generations eliminating the important confounding variable of mouse strain susceptibility to Bleomycin-induced lung fibrosis [25].

On day-21 after the single intratracheal administration of Bleomycin, we determined the degree of lung fibrosis in coded samples. This is a significant exposure for mice [18]. We evaluated lung fibrosis and fibrogenesis employing several complementary methods: i) trichrome stain; ii) Sirius red stain; iii) quantitative Sirius red collagen-binding assay [8],[26]; iv) quantitative hydroxyproline assay; v) quantitative collagen type 1 assay; vi) expression of α-smooth muscle actin (α-SMA) (present in activated LMF); and vii) expression of transforming growth factor (TGF)- β1 (a pro-fibrotic cytokine) [1],[5]–[7],[23],[27].

**Figure 1:**
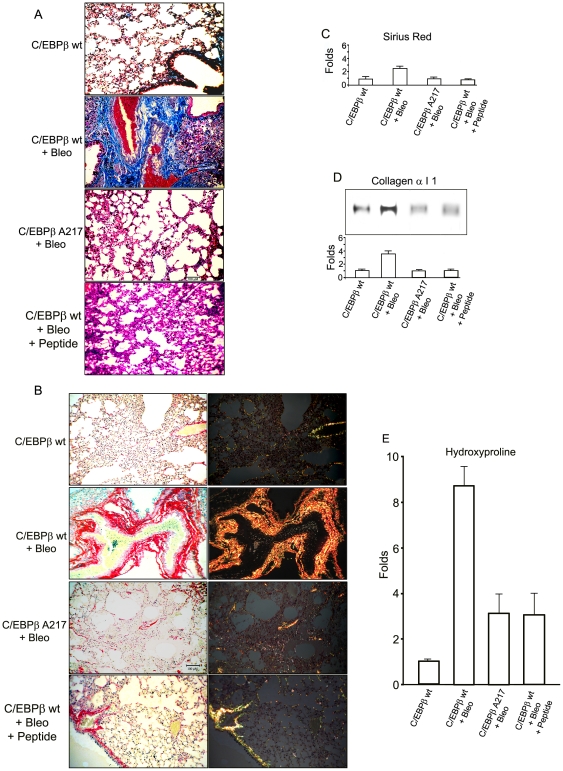
Blocking the C/EBPβ-Thr217 phosphorylation ameliorates the induction of lung fibrosis by Bleomycin. FVB C/EBPβ ^wt^ mice received once intracheally either Bleomycin or control saline as described in Methods. Analysis was performed on day-21. The bars represent 50 µm. **A.** Representative Mallory's trichrome stain for lung fibrosis (in blue). We graded the coded lung samples with the standard clinical system (0–3; none, mild, moderate and severe). After Bleomycin treatment, C/EBPβ^wt^ mice had moderate to severe lung fibrosis compared to controls, while C/EBPβ-Ala217 mice of the same genetic background had mild or moderate lung fibrosis (mean scores : 2.8+/−0.4 vs. 1.5+/−0.5; *n*: 10 per group; *P*<0.001). Treatment of C/EBPβ^wt^ mice with the C/EBPβ peptide (on day-2 and day-6) after Bleomycin treatment ameliorated the lung fibrosis at day-21 (mean fibrosis score: 2.0+/−0.7; *n*: 5; *P* = 0.048). **B.** Representative Sirius red immunohistochemistry for collagen by light microscopy (in red) (L panels) and with a polarized light (R panels). Increase in lung collagen in a fibrotic pattern was observed in C/EBPβ^wt^ but not in C/EBPβ-Ala 217 mice, treated with Bleomycin. Treatment of C/EBPβ^wt^ mice with the C/EBPβ peptide (on day-2 and day-6) after Bleomycin treatment decreased the lung fibrosis at day-21. **C, D and E.** Quantitation lung fibrosis was performed by the Sirius red collagen–binding, collagen type 1 and hydroxyproline assays as described in Methods. The lung collagen content increased (fold from baseline) in C/EBPβ^wt^ mice (*n*: 7 per group; Sirius red collagen-binding: 2.8+/−0.3; collagen type: 3.6+/−0.5; and hydroxyproline: 8.7+/−0.9; *P*<0.0001 for all), while remaining closer to baseline in C/EBPβ-Ala217 mice treated with Bleomycin (*n*: 6 ; Sirius red collagen-binding: 1.1+/−0.2, *P* = 0.37 ; collagen type: 1.0+/−0.2, *P* = 0.46; and hydroxyproline: 3.1+/−1.0, *P* = 0.002). The specific fold changes in C/EBPβ^wt^ mice treated with Bleomycin and the C/EBPβ peptide included: Sirius red collagen-binding (0.94+/−0.2, *P* = 0.80); collagen type 1 (1.1+/−0.2, *P* = 0.934); and hydroxyproline (3.1+/−1.0, *P* = 0.002; *n*: 5 for all groups). Representative results from three independent studies for the transgenic mice and of two independent studies for the peptide.

Lung samples were stained with the classical Mallory's trichrome to identify collagen in the extracellular matrix. As expected, the lung collagen pattern and content of C/EBPβ^wt^ mice on day-21 after Bleomycin treatment were increased similarly to those previously reported [9],[18]–[21],[25] (**Figure 1A**). We graded the coded lung samples with the standard clinical system (0–3; none, mild, moderate and severe), and found that after Bleomycin treatment, C/EBPβ^wt^ mice had moderate to severe lung fibrosis (mean score 2.8+/−0.4; *n*: 10) compared to controls , while C/EBPβ-Ala217 mice of the same genetic background had mild or moderate lung fibrosis (mean score: 1.5+/−0.5; *n*: 10; *P<*0.001; Wilcoxon U Test) (**Figure 1A**). In agreement with the findings with the Mallory's trichrome, the Sirius red collagen-binding stain under polarized light also demonstrated decreased lung fibrosis after Bleomycin treatment in C/EBPβ-Ala217 mice (**Figure 1B**). Although the intra- and inter-observer variability in the semi-quantitative analysis of lung fibrosis was low, we confirmed these findings using quantitative analysis of lung fibrosis.

The quantitative analysis of lung collagen, the major extracellular matrix protein in lung fibrosis [1], with assays for Sirius red collagen-binding, hydroxyproline and collagen type 1 demonstrated that C/EBPβ-Ala217 mice were refractory to the development of lung fibrosis after exposure to the lung toxin (**Figure 1C, 1D and 1E**). The lung collagen content increased approximately 2- to 8-fold from baseline in C/EBPβ^wt^ mice (*n*: 7 per group; Sirius red collagen-binding: 2.8+/−0.3; collagen type: 3.6+/−0.5; and hydroxyproline: 8.7+/−0.9; *P<*0.0001 for all), while remaining closer to baseline in C/EBPβ-Ala217 mice treated with Bleomycin (*n*: 6; Sirius red collagen-binding: 1.1+/−0.2, *P* = 0.37; collagen type: 1.0+/−0.2, *P* = 0.46; and hydroxyproline: 3.1+/−1.0, *P* = 0.002) (**Figure 1C, 1D and 1E**).

**Figure 2:**
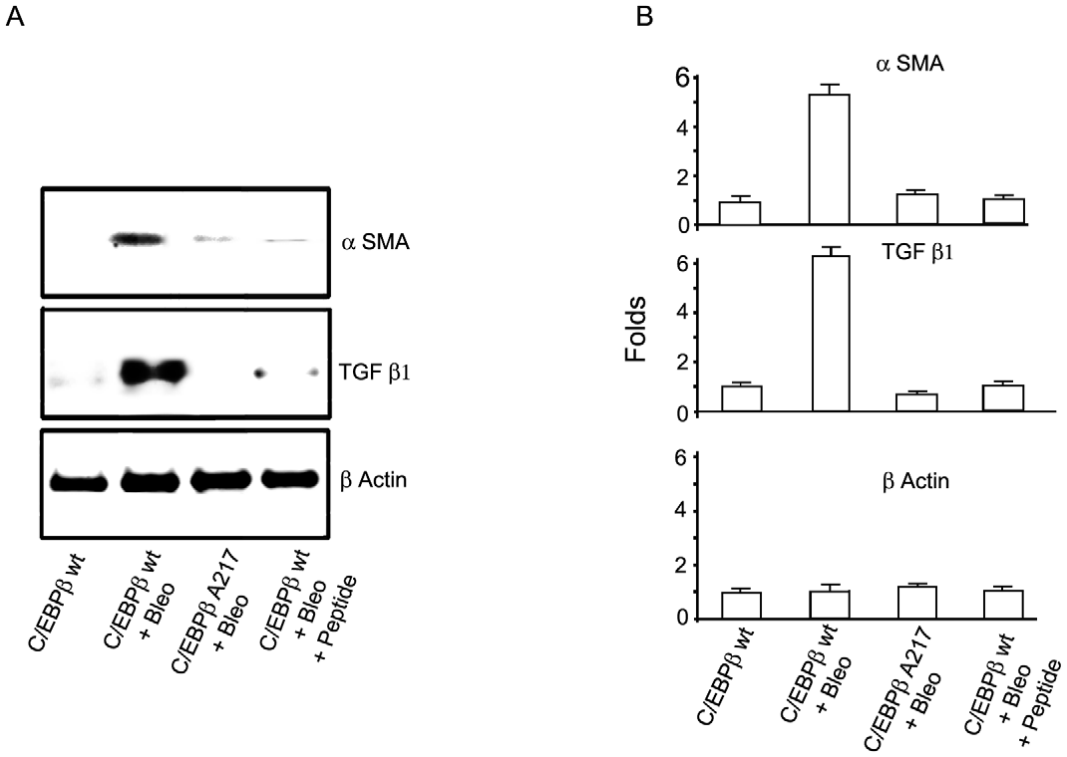
Blocking the C/EBPβ-Thr217 phosphorylation decreases the Bleomycin-induced activation of LMF. Mice were treated with Bleomycin or saline as described in Methods. Analysis was performed on day-21 **A**. Immunoblots for α-SMA, TGF-β and β-Actin were performed as described in Materials and methods. **B**. Pixel intensity was quantified by the Odyssey Western Infrared Detection Method and Odyssey Visualization protocol as described in Methods. The fold-increase in protein expression of lung fibrogenic indicators α-SMA and TGF-β1 were induced by Bleomycin in C/EBPβ^wt^ mice (*n*: 7 per group; α-SMA: 5.2+/−0.3, *P*<0.0001; TGF-β1: 5.9+/−0.3 *P*<0.0001) but not in C/EBPβ-Ala217 mice (*n*: 6 per group; α-SMA: 1.2+/−0.2, *P* = 0.15; TGF-β1 : 0.7+/−0.2 , *P* = 0.013) as measured by immunoblots Treatment of C/EBPβ^wt^ mice with the C/EBPβ peptide (on day-2 and day-6) after Bleomycin treatment also ameliorated the expression of α-SMA protein (1.0+/−0.18 , *P* = 0.428) and TGF-β1 protein (1.1+/−0.17, *P* = 0.793). β-Actin was used to correct for lung lysate input. Representative results from three independent studies.

Further, the protein expression of lung fibrogenic indicators α-SMA, present in activated myofibroblasts (NM_007392.2) and TGF-β1, a fibrogenic cytokine [27] (NM_009370.2) were induced by Bleomycin in C/EBPβ^wt^ mice ∼5-fold (*n*: 7 per group; α-SMA: 5.2+/−0.3, *P<*0.0001; TGF-β1: 5.9+/−0.3 *P<*0.0001) but not in C/EBPβ-Ala217 mice (*n*: 6 per group; α-SMA: 1.2+/−0.2, *P* = 0.15; TGF-β1: 0.7+/−0.2, *P* = 0.013) as measured by immunoblots (**Figure 2A and 2B**). Pixel intensity was quantified by the Odyssey Western Infrared Detection Method and Odyssey Visualization protocol as described previously [28] (**Figure 2B**). In summary, expression of the non-phosphorylatable, dominant negative C/EBPβ-Ala217 transgene decreases the fibrogenic response of the lung to Bleomycin -induced injury (**Figures 1** **and** **2**).

### Mice expressing the dominant negative C/EBPβ-Ala217 transgene are resistant to Bleomycin-induced lung injury

The degree of the initial lung injury, early inflammatory foci and bone marrow–derived cell activation contribute to the development of lung fibrosis [6],[7],[18],[19],[29]. Therefore, we assessed the degree of lung injury and inflammation in response to the lung toxin.

**Figure 3:**
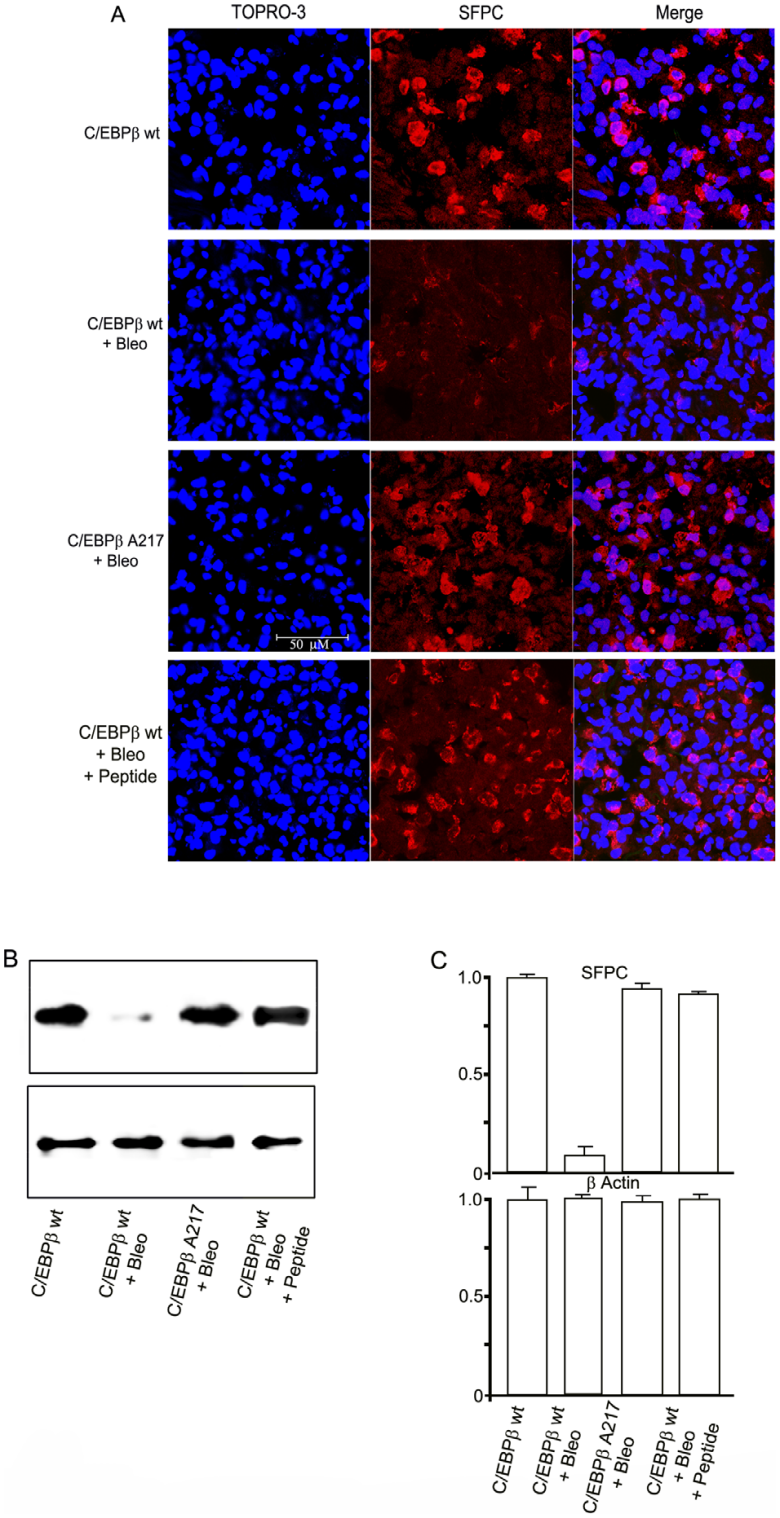
Mice expressing the C/EBPβ-Ala217 transgene are refractory to the induction of lung injury. C/EBPβ ^wt^ and C/EBPβ-Ala217 mice received once intracheally either Bleomycin or control saline as described in Methods. Analysis was performed on day-13. The bars represent 50 µm. **A.** Representative examples of the expression of Surfactant Protein C (SFPC), identified by confocal microscopy (red). It was decreased in the lungs of C/EBPβ ^wt^ mice treated with Bleomycin, but not in the lungs of C/EBPβ-Ala217 mice treated with Bleomycin or in the lungs of C/EBPβ ^wt^ treated with Bleomycin and the C/EBPβ peptide as described in Methods. Nuclei are identified with TO-PRO-3 (blue). Only background staining was observed when omitting the first antibody. **B.** Expression of SFPC as determined by immunoblots as described in Methods. **C.** Pixel intensity was quantified in a Kodak-4000 Imaging Station as described in Methods. C/EBPβ-Ala217 mice had much less lung injury than C/EBPβ^wt^ mice after Bleomycin treatment (n: 5 per group; 0.81+/−0.06 vs 0.04+/−0.25; *P* = 0.006). The expression of SFPC protein was decreased from baseline by Bleomycin treatment in both C/EBPβ^wt^ (*P* = 0.0003) and C/EBPβ-Ala217 mice (*P* = 0.0042). C/EBPβ^wt^ mice that received the C/EBPβ peptide had less lung injury than control C/EBPβ^wt^ mice after Bleomycin treatment, judging by the SFPC expression (n: 5; 0.78+/−0.05 vs 0.04+/−0.25; *P* = 0.008). The expression of SFPC protein was decreased from baseline by Bleomycin treatment in animals receiving the peptide (*P* = 0.0019). β-Actin was used to correct for lung lysate input. Representative results from two independent studies.

Decreased surfactant protein-C (SFPC) production by type II pneumocytes is a sensitive and specific indicator of alveolar epithelial lung injury in humans and animals [30],[31]. Therefore, we evaluated lung injury in mice on day-13 after exposure to Bleomycin by measuring SFPC lung expression. We found that C/EBPβ-Ala217 mice had much less lung injury than C/EBPβ^wt^ mice after Bleomycin treatment, judging by the SFPC protein expression as determined by confocal microscopy (**Figure 3A**) and quantitative immunoblots (n: 5 per group; 0.81+/−0.06 vs 0.04+/−0.25; *P* = 0.006) (**Figure 3B and 3C**). The expression of SFPC protein was decreased from baseline by Bleomycin treatment in both C/EBPβ^wt^ (*P* = 0.0003) and C/EBPβ-Ala217 mice (*P* = 0.0042) (**Figure 3B and 3C**).

**Figure 4:**
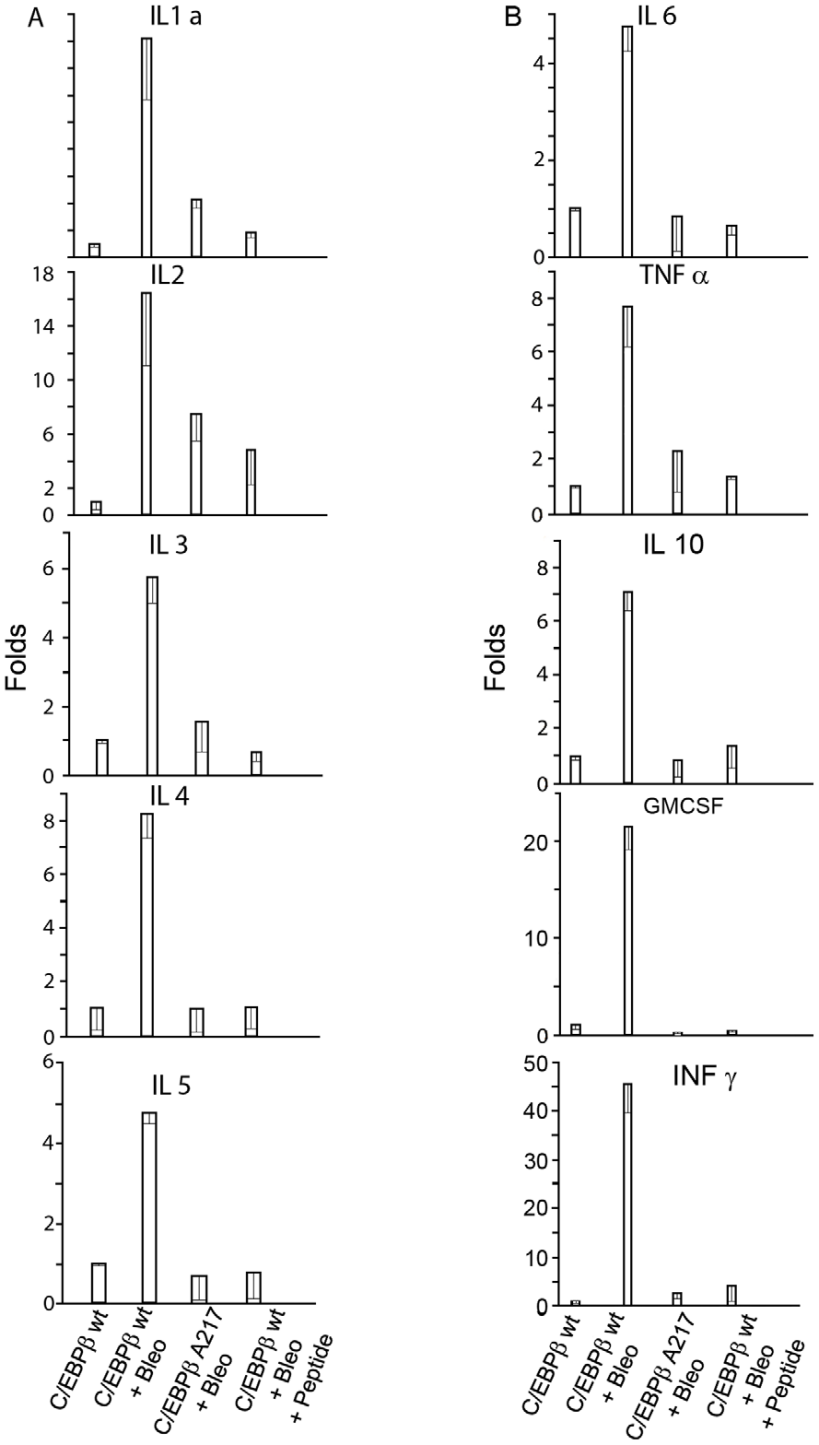
Mice expressing the C/EBPβ-Ala217 transgene are refractory to the induction of lung inflammation. Animals received either a single intratracheal instillation of Bleomycin or saline as described in Methods. On day-13 , Bleomycin induced several fold the protein expression of cytokines IL-1-α, IL-2, IL-3, IL-4, IL-5, IL-6, IL-10, TNF-α, IFN-γ and GM-CSF in the lungs of C/EBPβ^wt^ animals (*n:*3 per group; *P*<0.01 for all cytokines). The protein cytokine induction was lower in the lungs from C/EBPβ-Ala217 mice than in the lungs of C/EBPβ^wt^ mice on day-13 after Bleomycin–induced lung injury (*n:* 3 per group; *P*<0.01 for all cytokines, except for IL-2; *P* = 0.56). C/EBPβ^wt^ mice that received the C/EBPβ peptide had less lung inflammation than control C/EBPβ^wt^ mice after Bleomycin treatment (*n:* 3 per group; *P*<0.05 for all cytokines).

Because the C/EBPβ-Ala217 transgene inhibited lung injury (**Figure 3**), we determined whether lung cytokine protein expression is decreased in C/EBPβ-Ala217 mice. We used a multiplex nano-array assay to assess expression of ten inflammatory cytokine proteins in the lungs of control and transgenic mice. As expected, on day-13 Bleomycin induced several fold the protein expression of cytokines interleukin (IL-1)- α , IL-2, IL-3, IL-4, IL-5, IL-6, IL-10 , tumor necrosis factor (TNF)- α, interferon (IFN)-γ and granulocyte-macrophage colony-stimulating factor (GM-CSF) in the lungs of C/EBPβ^wt^ animals (*n:* 3 per group; *P<*0.01 for all cytokines) (**Figure 4**). The protein cytokine induction was lower in the lungs from C/EBPβ-Ala217 mice than in the lungs of control mice on day-13 after Bleomycin–induced lung injury (*n:* 3 per group; *P<*0.01 for all cytokines, except for IL-2; *P* = 0.56) (**Figure 4**).

In addition, a decreased inflammatory response, mediated at least in part by monocytes/macrophages in the lungs of C/EBPβ^wt^ mice may be responsible for the decreased lung injury in C/EBPβ-Ala217 mice treated with Bleomycin (**Figure S1**) since it also affected the recruitment to the lung of CD68^+^ activated monocytes/macrophages and bone marrow-derived CD45^+^ cells (**Figures S2 and S3**). These data suggest that partial resistance to lung injury and inflammation may contribute to the prevention of lung fibrosis in C/EBPβ-Ala217 mice.

### Mice expressing the dominant negative C/EBPβ-Ala217 transgene are resistant to Bleomycin-induced LMF activation

Quiescent resident lung or bone marrow-derived fibroblasts produce negligible amounts of ECM, but after their activation, these cells develop a myofibroblast phenotype, proliferate and become the main contributors of ECM [1],[6],[7],[19]. Because this step may facilitate development of lung fibrosis foci [32], we analyzed the activation and proliferation of LMF in the lungs of mice on day-21 after exposure to Bleomycin.

**Figure 5:**
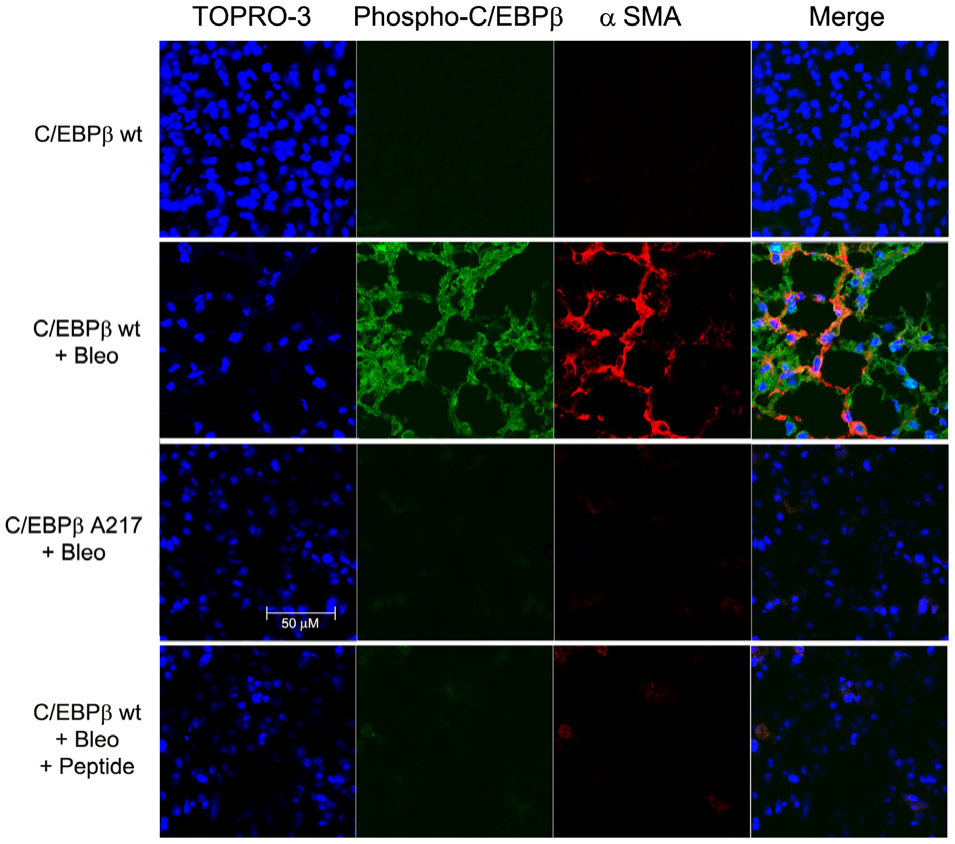
Mice expressing the C/EBPβ-Ala217 transgene are refractory to lung myofibroblast activation. Mice were treated with Bleomycin as described in Materials and methods. Analysis was performed on day-21 The bars represent 50 µm. **A.** Activated LMF, identified by confocal microscopy for α-smooth muscle actin (α-SMA; red), displayed C/EBPβ-PhosphoThr217 (green) in lungs of C/EBPβ ^wt^ treated with Bleomycin, but not in lungs of C/EBPβ-Ala217 mice treated with Bleomycin or in the lungs of C/EBPβ ^wt^ treated with Bleomycin and the C/EBPβ peptide. Co-localization of α-SMA and C/EBPβ-PhosphoThr217 is shown in yellow (merge). Nuclei are identified with TO-PRO-3 (blue). Only background staining was observed when omitting the first antibody.

As expected, Bleomycin administration to C/EBPβ^wt^ mice, induced marked activation of LMF, as indicated by the positive immunofluorescence for α-SMA within the scar tissue [19] (**Figure 5**). By contrast, C/EBPβ-Ala217 mice were refractory to the induction of LMF activation by Bleomycin treatment (**Figure 5**).

As determined by scanning confocal microscopy, C/EBPβ phosphorylation was negligible in lungs of untreated C/EBPβ^wt^ mice but it was induced by Bleomycin in these animals but not in C/EBPβ-Ala217 mice (**Figures 5** **and** **6**), suggesting that C/EBPβ expression and phosphorylation are associated with activation of LMF and lung injury. As expected, phosphorylation of C/EBPβ-Thr217 in LMF (α-SMA^+^) was induced by Bleomycin in C/EBPβ^wt^ mice but not in C/EBPβ-Ala217 mice (**Figure 5**). The phospho-C/EBPβ-Thr217 was mainly nuclear but also cytoplasmic. The cytoplasmic localization is most likely related to an additional C/EBPβ phosphorylation on Ser239, within the DNA-binding domain, that we have previously characterized [33]. This C/EBPβ-Ser239 phosphorylation , which is induced through oxidative stress and NO , neutralizes the nuclear localization signal and facilitates the interaction of the nuclear export signal with the nucleo-cytoplasmic shuttle protein CRM1 [34].

**Figure 6:**
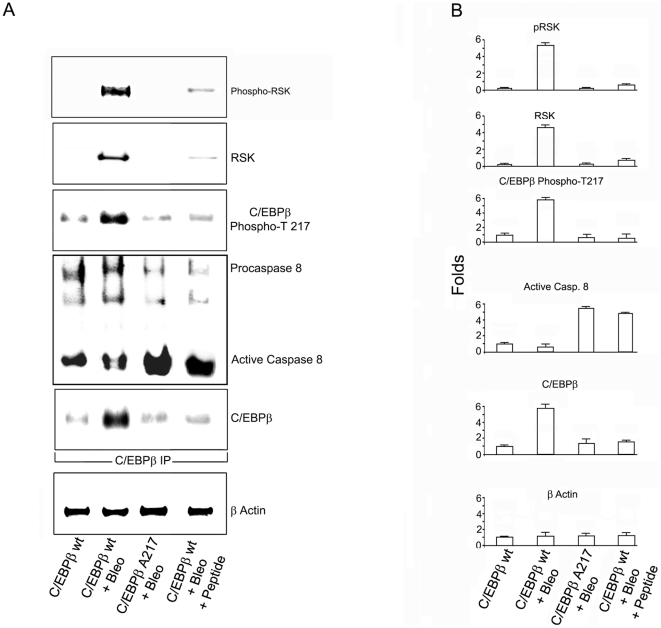
Inhibition of the RSK signaling prevents C/EPBβ phosphorylation and stimulates the association of unphosphorylated C/EBPβ with active caspase 8 in Bleomycin-induced lung injury. **A.** Immunoblots for phospho-RSK, RSK, C/EBPβ-phospho-Thr217, procaspase 8 and C/EBPβ were performed on C/EBPβ immunoprecipitates from lung protein lysates in an experiment conducted as described in (Fig. 1). Phospho-RSK, RSK and phosphorylated C/EBPβ were increased in C/EBPβ immunoprecipitates in lungs of C/EBPβ^wt^ treated with Bleomycin but not in C/EBPβ-Ala217 mice treated with Bleomycin or in the lungs of C/EBPβ^wt^ treated with Bleomycin and the C/EBPβ peptide. Active caspase 8 association with unphosphorylated C/EBPβ increased in C/EBPβ-Ala217 mice treated with Bleomycin and in C/EBPβ ^wt^ mice treated with Bleomycin and the C/EBPβ peptide. C/EBPβ was expressed as C/EBPβΔ154 also known as LIP. β-Actin was used to correct for lung lysate input. **B.** Pixel intensity was quantified by the Odyssey Western Infrared Detection Method and Odyssey Visualization protocol as described in Methods. After Bleomycin administration, C/EBPβ and phosphorylated C/EBPβ-Thr217 were induced several fold in the lungs of C/EBPβ^wt^ mice (n: 6 per group; C/EBPβ: 5.56+/−0.52, *P*<0.0001; phosphorylated C/EBPβ-Thr217: 5.48+/−0.37, *P*<0.0001), but not in the lungs of C/EBPβ-Ala217 mice (n: 6 per group; C/EBPβ: 1.20+/−0.11, *P* = 0.332; phosphorylated C/EBPβ-Thr217: 1.10+/−0.17, *P* = 0.510). There was an increased fold association between RSK and phosphorylated, activated RSK with phosphorylated C/EBPβ-Thr217 in lungs of C/EBPβ^wt^ mice treated with Bleomycin (n: 6 per group; RSK: 4.85+/−0.31, *P*<0.0001; phosphorylated, activated RSK: 5.07+/−0.37, *P*<0.0001) but not C/EBPβ-Ala217 mice treated with Bleomycin (n: 6 per group; RSK: 0.87+/−0.12, *P* = 0.242; phosphorylated, activated RSK: 1.01+/−0.21, *P* = 0.633). The fold association between C/EBPβ with active caspase 8 was greater in the lungs from C/EBPβ-Ala217 mice after Bleomycin administration (n: 6 per group; active caspase 8: 5.65+/−0.40, *P*<0.0001) than in the lungs from C/EBPβ^wt^ mice after Bleomycin administration (n: 6 per group; active caspase 8: 0.99+/−0.12, *P* = 0.362). After Bleomycin administration, mice that received the C/EBPβ peptide had a lower fold induction than control C/EBPβ^wt^ mice after Bleomycin treatment in the expression of the following lung protein: (n: 7 , controls; n:6, Bleomycin and n:5, peptide ; C/EBPβ: 1.23+/−0.39, *P* = 0.479 vs controls and *P* = 0.0001 vs. Bleomycin; phosphorylated C/EBPβ-Thr217: 1.03+/−0.18 , *P* = 0.258 vs. controls and *P* = 0.0001 vs. Bleomycin). There was an increased fold association between RSK and phosphorylated, activated RSK with phosphorylated C/EBPβ-Thr217 in lungs of C/EBPβ^wt^ mice treated with Bleomycin and the peptide (n: 7, controls; n:6, Bleomycin and n:5, peptide; RSK: 2.17+/−0.22, *P*<0.0001 vs. control and *P*<0.0001 vs Bleomycin ; phosphorylated, activated RSK: 1.44+/−0.22, *P*<0.170 vs. controls and *P*<0.0001 vs Bleomycin). The fold association between C/EBPβ with active caspase 8 was greater in the lungs from C/EBPβ^wt^ mice treated with the Ac-KAla217VD-CHO peptide after Bleomycin administration (n: 5; active caspase 8: 5.65+/−0.40, *P*<0.0001) that in the lungs from C/EBPβ^wt^ mice after Bleomycin administration.

**Figure 7:**
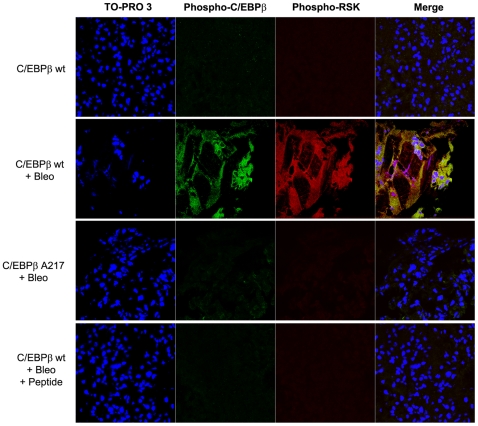
Induction and co-localization of active RSK and C/EBPβ-PhosphoThr217 in Bleomycin-induced lung fibrosis. Mice were treated with Bleomycin as described in (Fig. 1) and confocal microscopy was performed as described in Methods. Activated RSK-PhosphoSer380 (red) and C/EBPβ-PhosphoThr217 (green) were induced and co-localized at day-21 in the lungs of C/EBPβ ^wt^ treated with Bleomycin, but not in C/EBPβ-Ala217 mice treated with Bleomycin or in the lungs of C/EBPβ ^wt^ treated with Bleomycin and the C/EBPβ peptide. Nuclei are identified with TO-PRO-3 (blue). Only background staining was observed when omitting the first antibody. The bar represents 50 µm.

We analyzed immunoblots from C/EBPβ immunoprecipitates of lung lysates (**Figure 6A and 6B**) using specific antibodies [8],[10]. Pixel intensity was quantified by the Odyssey Western Infrared Detection Method and Odyssey Visualization protocol as described previously [28] (**Figure 6B**). After Bleomycin administration, C/EBPβ and phosphorylated C/EBPβ-Thr217 were induced several fold in the lungs of C/EBPβ^wt^ mice (n: 6 per group; C/EBPβ: 5.56+/−0.52, *P<*0.0001; phosphorylated C/EBPβ-Thr217: 5.48+/−0.37 , *P<*0.0001), but not in the lungs of C/EBPβ-Ala217 mice (n: 6 per group; C/EBPβ: 1.20+/−0.11, *P* = 0.332; phosphorylated C/EBPβ-Thr217: 1.10+/−0.17, *P* = 0.510). These studies also demonstrated the increased fold association between RSK and phosphorylated, activated RSK with phosphorylated C/EBPβ-Thr217 in lungs of C/EBPβ^wt^ mice treated with Bleomycin (n: 6 per group; RSK: 4.85+/−0.31, *P<*0.0001; phosphorylated, activated RSK: 5.07+/−0.37, *P<*0.0001) but not C/EBPβ-Ala217 mice treated with Bleomycin (n: 6 per group; RSK: 0.87+/−0.12, *P* = 0.242; phosphorylated, activated RSK: 1.01+/−0.21, *P* = 0.633) (**Figure 6B**). Phosphorylated, activated RSK was co-localized with phosphorylated C/EBPβ-Thr217 by confocal microscopy in lungs of C/EBPβ^wt^ mice treated with Bleomycin but not in the lungs of C/EBPβ-Ala217 mice treated with Bleomycin (**Figure 7**). C/EBPβ was induced in C/EBPβ^wt^ mice by Bleomycin as C/EBPβΔ154 (LIP) an alternative, shorter translation product initiated at the third AUG codon of the C/EBPβ mRNA, that shares with C/EBPβ the Thr-217 phosphoacceptor, the DNA binding and leucine zipper domains [35]. The expression of full-length C/EBPβ from the second AUG in the lungs of C/EBPβ^wt^ mice and C/EBPβ-Ala217 transgene was minimal at baseline and not induced by Bleomycin treatment.

### Unphosphorylated C/EBPβ-Thr217 is associated with members of the active caspase 8 death receptor complex II in the lungs of Bleomycin -treated animals

We found that the fold association between C/EBPβ with active caspase 8 was greater in the lungs from C/EBPβAla217 mice after Bleomycin administration (n: 6 per group; active caspase 8: 5.65+/−0.40, *P<*0.0001) that in the lungs from C/EBPβ^wt^ mice after Bleomycin administration (n: 6 per group; active caspase 8: 0.99+/−0.12, *P* = 0.362) (**Figure 6A and 6B**). The increased activation of initiator caspase 8 (**Figure 6**) , and RIP, a member of the caspase 8 complex II (**Figure S4**), in the lungs of the nonphosphorylatable C/EBPβ-Ala217 mice after Bleomycin administration is congruent with our report that phospho-C/EBPβ-Thr217 inhibits procaspase 8 activation, and that blocking this site-specific phosphorylation results in activation of caspase 8 [10],[36].

### Unphosphorylated C/EBPβ-Thr217 is associated with active caspase 8 and induction of cell death pathways in activated human lung fibroblasts

Because the mixed cell population of the lung limits the evaluation of signaling cascades in a specific cell type, we studied the fibrogenic pathway in cultured primary human lung fibroblasts. The lung fibroblasts were activated on collagen type 1, a condition that recapitulates the activation of LMF in vivo, and identified by their expression of α-SMA [1],[6],[7],[19]. Because myofibroblasts from IPF patients are already abnormally activated and they would not allow analysis of the transition into activated LMF [1],[6],[7],[19], as a proof of principle we used lung fibroblasts derived from normal lungs. A similar strategy using normal, quiescent myofibroblasts has been effective in developing activated myofibroblasts from the liver and other tissues [8],[10],[23].

**Figure 8:**
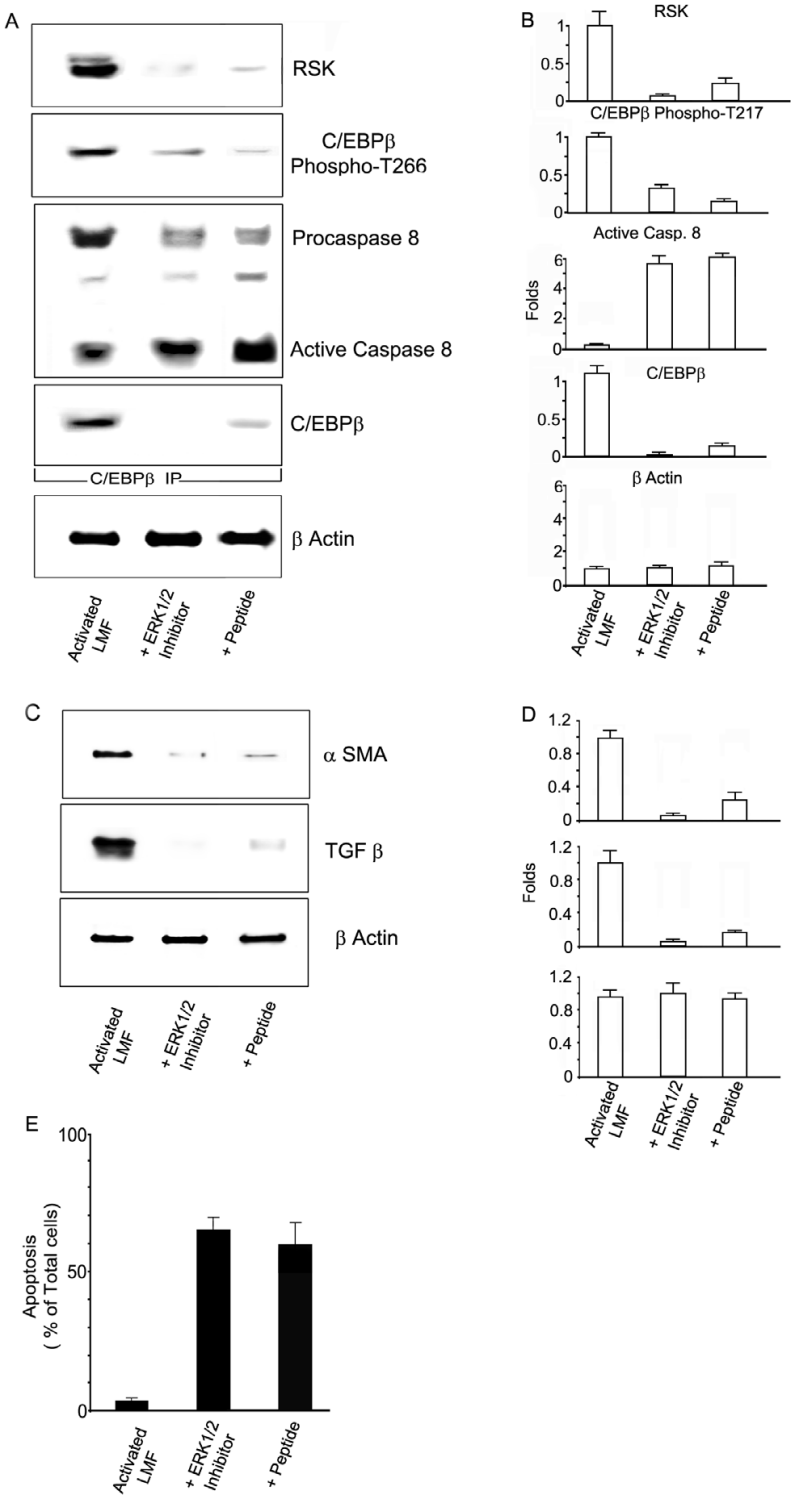
Inhibition of the RSK signaling prevents C/EPBβ phosphorylation and stimulates apoptosis in activated lung myofibroblasts. **A.** Immunoblots for RSK, C/EBPβ-phospho-Thr217, procaspase 8 and C/EBPβ were performed on C/EBPβ immunoprecipitates from activated primary human LMF lysates as described in Methods. RSK and phosphorylated C/EBPβ were induced in activated LMF but decreased in activated LMF treated with the ERK1/2 inhibitor (10 µg for 24 hr) or with the C/EBPβ peptide (200 µg for 24 hr). Inactive procaspase 8 was associated with phosphorylated C/EBPβ in untreated, activated LMF, while active caspase 8 was associated with unphosphorylated C/EBPβ in activated LMF treated with the ERK1/2 inhibitor or with the C/EBPβ peptide. Human LMF expressed full-length C/EBPβ from the second AUG [35]. β-Actin was used to correct for lung lysate input. We performed single analysis of the samples. Results from triplicate samples of two independent experiments are shown. **B.** The ERK1/2 inhibitor decreased the fold association of RSK with C/EBPβ (n: 6 per group; 0.07+/−0.01, *P*<0.0001), and the fold expression of C/EBPβ (n: 6 per group; 0.05+/−0.006, *P*<0.0001) and C/EBPβ-PhosphoThr266 (n: 6 per group; 0.33+/−0.078, *P*<0.0001) in activated, human lung fibroblasts. There was also an increased fold association between unphosphorylated human C/EBPβ and active caspase 8 (n: 6 per group; active caspase 8; 5.70+/−0.59, *P*<0.0001). The cell permeant Ac-KAla217VD-CHO peptide also inhibited C/EBPβ expression (n: 6 per group; 0.12+/−0.01, *P*<0.0001), the phosphorylation of C/EBPβ-Thr266 (n: 6 per group; 0.17+/−0.03, *P*<0.0001), the association of RSK with C/EBPβ (n: 6 per group; 0.24+/−0.08, *P*<0.0001) and increased the association of RSK with C/EBPβ (n: 6 per group; 0.24+/−0.08, *P*<0.0001). We performed single analysis of the samples. **C.** α-SMA and TGF-β were induced in activated LMF but the expression of these fibrogenic genes was inhibited by treatment with the ERK1/2 inhibitor (10 µg for 24 hr) or with the peptide (200 µg for 24 hr). β-Actin was used to correct for lung lysate input. Representative results from two independent studies. **D.** The ERK1/2 inhibitor decreased the fold expression of α-SMA (n: 6 per group; 0.24+/−0.11, *P*<0.0001) and TGF-β1 (n: 6 per group; 0.14+/−0.02, *P*<0.0001). The cell permeant Ac-KAla217VD-CHO peptide also inhibited the fold expression of α-SMA (n: 6 per group; 0.24+/−0.11, *P*<0.0001) and TGF-β1 (n: 6 per group; 0.14+/−0.02, *P*<0.0001). We performed single analysis of the samples. **E.** Annexin-V-PE binding in vivo in activated LMF was increased after treatment with the ERK1/2 inhibitor (20 µg for 8 hr) or with the peptide (200 µg for 24 hr). Values are the percentage of cells expressing annexin-V-PE binding as described in Methods. Activated, human lung fibroblasts treated with the ERK1/2 inhibitor (n: 6; 66.33+/−5.68%, *P*<0.0001) or with the Ac-KAla217VD-CHO peptide (n: 6; 61.00+/−9.27%, *P*<0.0001) displayed increased percent annexin-V binding compared to control (n: 6; 4.15+/−0.94%). We performed single analysis of the samples. Results from triplicate samples of three independent experiments are shown.

We found that an ERK1/2 inhibitor that blocks the MAPK signaling immediately upstream of RSK and prevents C/EBPβ-Thr266 phosphorylation (identical to mouse phosphoacceptor Thr217) [8], decreased the fold expression of RSK (n:6 per group ; 0.07+/−0.01, *P<*0.0001), C/EBPβ (n: 6 per group; 0.05+/−0.006, *P<*0.0001) and C/EBPβ-PhosphoThr266 (n: 6 per group; 0.33+/−0.078, *P<*0.0001) in activated, human lung fibroblasts (**Figure 8A and 8B**). Moreover, in these activated, human lung fibroblasts treated with the ERK1/2 inhibitor, there was also an increased fold association between unphosphorylated human C/EBPβ (NP_005185) and active caspase 8 (n: 6 per group; active caspase 8: 5.70+/−0.59 , *P<*0.0001) (**Figure 8A and 8B**). In contrast, in activated, human lung fibroblasts untreated with the ERK1/2 inhibitor, C/EBPβ-Thr266 was phosphorylated and associated with procaspase 8 (**Figure 8A and 8B**). The RSK phosphoacceptor site in human C/EBPβ-Thr266 appears to be essential for preventing activation of death cascades in lung fibroblasts upon their activation. The cultured human lung fibroblasts expressed mainly C/EBPβΔ154 and to a lesser degree full-length C/EBPβ translated from the second AUG [35].

Further, the ERK1/2 inhibitor decreased the fold expression of protein indicators of activated, human lung fibroblasts as measured by quantitative immunoblots (*n*: 6 per group; α-SMA: 0.05+/−0.03, *P<*0.0001; TGF-β1: 0.03+/−0.01 *P<*0.0001) (**Figure 8C and 8D**). In addition, activated, human lung fibroblasts treated with the ERK1/2 inhibitor displayed increased percent annexin-V binding to phosphatidylserine in plasma membranes (n: 6 per group; annexin-V binding: 4.15+/−0.94% vs 66.33+/−5.68%, *P<*0.0001), an early indicator of apoptosis [10] (**Figure 8E**).

In summary, blocking phosphorylation of human C/EBPβ-Thr266 through the inhibition of the MAPK signaling upstream of RSK with the ERK1/2 inhibitor decreases the fibrotic response (**Figure 8C and 8D**) and induces the apoptotic program (**Figure 8A, 8B and 8E**) in activated primary human lung fibroblasts.

Next, we assessed whether the cell permeant Ac-KAla217VD-CHO peptide that inhibits phosphorylation of human C/EBPβ-Thr266 [8],[10] would have similar effects that the ERK1/2 inhibitor on activated, normal human lung fibroblasts. As we found with the ERK1/2 inhibitor, the peptide also inhibited C/EBPβ expression (n: 6 per group; 0.12+/−0.01, *P<*0.0001), the phosphorylation of C/EBPβ-Thr266 (n: 6 per group; 0.17+/−0.03 , *P<*0.0001), the association of RSK with C/EBPβ (n: 6 per group; 0.24+/−0.08, *P<*0.0001) (**Figure 8A and 8B**), the expression of α-SMA (n: 6 per group; 0.24+/−0.11, *P<*0.0001) and TGF-β1 (n: 6 per group; 0.14+/−0.02, *P<*0.0001) (**Figure 8C and 8D**), but increased the association of active caspase 8 with C/EBPβ (n: 6 per group; 6.15+/−0.27, *P<*0.0001) (**Figure 8A and 8B**) as well as the induction of apoptosis (n: 6 per group; 4.15+/−0.94% vs 61.00+/−9.27%, *P<*0.0001) (**Figure 8E**).

### A dominant negative C/EBPβ peptide inhibits progression of Bleomycin-induced lung injury and fibrosis

Given the effective blocking of molecular pathways leading to lung fibrosis in a Bleomycin model of lung injury and fibrosis by the dominant negative C/EBPβ-Ala217 transgene (**Figures 1** **,** **2** **and** **3**) and the anti-fibrogenic effects of a dominant negative C/EBPβ peptide on cultured human lung fibroblasts (**Figure 8**), we asked whether administration of the peptide could ameliorate the lung injury and fibrosis induced by Bleomycin.

We have reported that the cell permeant Ac-KAla217VD-CHO associates directly with procaspase 8 accelerating it self-processing at picomolar concentrations [8],[10] and that 1 to 100 µg of the cell permeant Ac-KAla217VD-CHO peptide administered intraperitoneally provided adequate systemic bioavailability with no detectable toxicity in mice [8]. Therefore, as a proof of principle we tested the effects of administering 40 µg of the peptide by intratracheal instillation on day-2 and day-6 after receiving Bleomycin on lung injury and fibrosis; more frequent intratracheal instillations in Bleomycin-treated mice were not well tolerated.

Treatment of C/EBPβ^wt^ mice with the peptide after receiving Bleomycin on day-0, prevented progression of lung fibrosis compared to control mice treated only with Bleomycin. At day-21, there was a marked inhibition of lung fibrosis judging by the trichrome stain and Sirius red stain (**Figure 1A and 1B**). Control mice had moderate to severe fibrosis (mean fibrosis score: 2.8+/−0.4; *n*: 10) while mice that received the C/EBPβ peptide had a moderate degree of lung fibrosis (mean fibrosis score: 2.0+/−0.7; *n*: 5; *P* = 0.048; Wilcoxon U Test).

We confirmed these findings by quantitative analysis of lung collagen with the Sirius red, collagen type 1 and hydroxyproline assays [8]. Lung collagen content increased approximately 2- to 8-fold from baseline in C/EBPβ^wt^ mice treated with Bleomycin while remaining closer to baseline in C/EBPβ^wt^ mice treated with Bleomycin and the C/EBPβ peptide (*P<*0.001 for all measurements). The specific fold changes in C/EBPβ^wt^ mice treated with Bleomycin and the C/EBPβ peptide included : Sirius red collagen-binding (0.94+/−0.2, *P* = 0.80); collagen type 1 (1.1+/−0.2, *P* = 0.934); and hydroxyproline (3.1+/−1.0, *P* = 0.002; *n*: 5; for all groups) (**Figure 1C, 1D and 1E**).

In agreement with the results observed after Bleomycin treatment in mice expressing the C/EBPβ-Ala217 transgene, treatment of C/EBPβ^wt^ mice with the C/EBPβ peptide ameliorated other effects of Bleomycin and it was similar to control untreated with Bleomycin: i) expression of lung fibrogenic indicators such as α-SMA protein (1.0+/−0.18, *P* = 0.428), and TGF-β1 protein (1.1+/−0.17, *P* = 0.793) (**Figure 2**); and ii) recruitment of CD68^+^ monocytes/macrophages and CD45^+^ bone marrow-derived fibrocytes to the lung (**Figure S2 and S3**).

Also we found that C/EBPβ^wt^ mice that received the C/EBPβ peptide had less lung injury than control C/EBPβ^wt^ mice after Bleomycin treatment, judging by the SFPC expression by confocal microscopy (**Figure 3A**) and quantitative immunoblots (n: 5; 0.78+/−0.05 vs 0.04+/−0.25; *P* = 0.008) (**Figure 3B and 3C**). The expression of SFPC protein was decreased from baseline by Bleomycin treatment in animals receiving the peptide (*P* = 0.0019) (**Figure 3B and 3C**).

Similarly, we found that the protein expression of cytokines interleukin (IL-1)-α, IL-2, IL-3, IL-4, IL-5, IL-6, IL-10, TNF-α, IFN-γ and GM-CSF was lower in the lungs from C/EBPβ^wt^ mice that received the C/EBPβ peptide than in the lungs from control C/EBPβ^wt^ mice after Bleomycin treatment (*n:*3 per group; *P<*0.05 for all cytokines) (**Figure 4**).

After Bleomycin administration, mice that received the C/EBPβ peptide had a much lower fold induction than control C/EBPβ^wt^ mice after Bleomycin treatment in the expression of the following lung protein: (n:7, controls; n:6, Bleomycin and n:5, peptide; C/EBPβ: 1.23+/−0.39, *P* = 0.479 vs controls and *P* = 0.0001 vs Bleomycin; phosphorylated C/EBPβ-Thr217: 1.03+/−0.18, *P* = 0.258 vs controls and *P* = 0.0001 vs Bleomycin) (**Figure 6**). These studies also demonstrated the increased fold association between RSK and phosphorylated, activated RSK with phosphorylated C/EBPβ-Thr217 in lungs of C/EBPβ^wt^ mice treated with Bleomycin and the peptide (n:7, controls; n:6, Bleomycin and n:5 , peptide ; RSK: 2.17+/−0.22, *P<*0.0001 vs control and *P<*0.0001 vs Bleomycin ; phosphorylated, activated RSK: 1.44+/−0.22, *P<*0.170 vs controls and *P<*0.0001 vs Bleomycin) (**Figure 6**). Phosphorylated, activated RSK was co-localized with phosphorylated C/EBPβ-Thr217 by confocal microscopy in lungs of C/EBPβ^wt^ mice treated with Bleomycin but not in the lungs of these animals when they received also treatment with the peptide (**Figure 7**).

We found that the fold association between C/EBPβ with active caspase 8 was greater in the lungs from C/EBPβ^wt^ mice treated with the Ac-KAla217VD-CHO peptide after Bleomycin administration (n: 5; active caspase 8: 5.65+/−0.40, *P<*0.0001) that in the lungs from C/EBPβ^wt^ mice after Bleomycin administration (n: 6; active caspase 8: 0.99+/−0.12, *P* = 0.362) (**Figure 6**). Also we found increased protein expression of RIP, a member of the caspase 8 complex II in the lungs of C/EBPβ^wt^ mice treated with the peptide mice after Bleomycin administration (**Figure S4**).

### Increased expression of active RSK and C/EBPβ-PhosphoThr266 in activated LMF of human lung fibrosis

**Figure 9:**
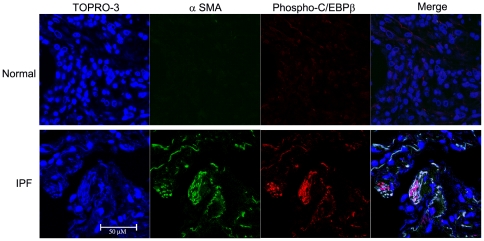
Increased expression of C/EBPβ-PhosphoThr266 in activated lung myofibroblasts of human lung fibrosis. Representative confocal microscopy of 2 IPF patients with severe lung fibrosis and 2 matched control subjects. C/EBPβ-PhosphoThr266 (in red) and α-SMA (in green) were present in activated LMF only in lungs of patients with lung fibrosis (lower panel). Co-localization of C/EBPβ-PhosphoThr217 and α-SMA is shown in white/yellow (merge). Nuclei are identified with TO-PRO-3 (blue). The bar represents 50 µm.

To assess the relevance to human lung fibrosis of the cellular and animal models of liver fibrosis, we analyzed, in preliminary studies, the role of activated RSK and phosphorylated C/EBPβ on Thr266 (identical to mouse Thr217 phosphoacceptor) as possible mechanisms leading to increased lung fibrosis in two patients with severe idiopathic lung fibrosis. Lung biopsies from patients afflicted with severe Idiopathic Pulmonary Fibrosis displayed a high level expression of both active, phosphorylated human C/EBPβ on Thr266 and phosphorylated RSK (that co-localized in activated α-SMA^+^ LMF within the fibrous tissue) (**Figures 9** **and S5**), compared with samples from two matched control patients as identified by confocal scanning microscopy with specific antibodies against RSK-PhosphoSer380, C/EBPβ-PhosphoThr266, and α-SMA for LMF [1].[7],[10]. Most of the activated RSK-PhosphoSer380 appears to be nuclear as reported previously for activated liver myofibroblasts [10] (**Figure S5**).

Thus, our findings in lung biopsies from patients with lung fibrosis are congruent with the results from cellular and animal models indicating that RSK induction and its phosphorylation of mouse C/EBPβ-Thr217 or human C/EBPβ-Thr266 in activated lung fibroblasts may be important in the development of human lung injury and fibrosis.

## Discussion

In this study, we have identified part of the molecular signaling responsible for the decreased lung fibrogenic response to Bleomycin reported in C/EBPβ^ko^ mice [9]. Collectively, our findings strongly suggest, but do not definitively prove, that the RSK phosphoacceptor site KAKKThrVDK in C/EBPβ , which is identical in mouse-Thr217 and human-Thr266 [15], is critical for lung injury and fibrogenesis in mice. This study also suggests that blocking the RSK-C/EBPβ-Thr217 phosphorylation pathway with either a single point mutation (Ala217), dominant negative transgene or a blocking peptide containing the mutated phosphoacceptor ameliorates the progression of lung injury and fibrosis induced by Bleomycin in mice. Inhibition C/EBPβ-Thr217 phosphorylation appears to affect lung fibrogenesis directly, by inhibiting LMF activation and indirectly, by reducing lung injury. These results are novel for lung injury and fibrosis and congruent with the role of the RSK-C/EBPβ-Thr217 phosphorylation pathway in liver fibrogenesis that we proposed [8],[10],[37].

The lung toxin Bleomycin induced severe lung fibrosis in C/EBPβ^wt^ mice but not in mice expressing C/EBPβ-Ala217, a non-phosphorylatable transgene, as detected by several complementary morphological, semi quantitative and quantitative assays. Blocking phosphorylation of C/EBPβ-Thr217 with the C/EBPβ-Ala217 transgene (**Figures 5** **and** **6**) was associated with a decrease in the lung fibrotic response to Bleomycin (**Figure 1**) and with a decreased expression of the lung fibrogenic indicators α-SMA (expressed in activated LMF) and TGF-β1 (a fibrogenic cytokine) [7],[27] (**Figure 2**). Given the potential confounding effects of strain variation on the development of pulmonary fibrosis induced by a single intratracheal administration of Bleomycin [25], all mice used in this study were of identical genetic background and back-crossed to the parental wild-type inbreed FVB mice for at least 10 generations.

Because lung injury and inflammation may induce lung fibrosis [1],[7] we assessed the degree of lung injury induced by Bleomycin. In response to Bleomycin treatment, on day-13 we found decreased lung injury (as assessed by SFPC protein expression in the lungs) and inflammation (as assessed by the presence of CD68^+^ and CD45^+^ cells as well as cytokine protein expression in the lungs) in mice expressing the C/EBPβ-Ala217 transgene (**Figures 3** **, S2, S3 and** **4**). Our results suggest that resistance to lung injury may contribute to the prevention of lung fibrosis in C/EBPβ-Ala217 mice and are consistent with the proposed role of lung injury in the development of lung fibrosis [7],[30]. Indeed, inhibition of SFPC production by type II pneumocytes is a sensitive and specific indicator of alveolar epithelial lung injury in humans and animals [30],[31]. Some familial forms of lung fibrosis are associated with mutations in the SFPC gene [38], and mice lacking SFPC had greater lung inflammation and fibrosis after Bleomycin treatment [39]. Further, surfactant administration including SFPC to the lungs has dramatically improved the lung injury and inflammation of preterm infants with respiratory distress syndrome [40]. We have shown that blocking the phosphorylation of C/EBPβ-Thr217 by expressing the C/EBPβ-Ala217 transgene during a toxic injury induces apoptosis of activated liver myofibroblasts [8],[10] and of activated lung myofibroblasts (**Figures 6** **and** **8**) but not of hepatocytes [8],[10] or pneumocytes (**Figure 3**) . This effect is most likely related to the check-point of activated myofibroblasts since neither quiescent liver myofibroblasts nor hepatocytes are affected by the induction of apoptosis [10].

We have reported that the cell permeant, dominant negative C/EBPβ peptide, Ac-KAla217VD-CHO, associates directly with procaspase 8 accelerating it self-processing at picomolar concentrations [8],[10]. We have also reported that 1 to 100 µg of the cell permeant Ac-KAla217VD-CHO peptide administered intraperitoneally provided adequate systemic bioavailability with no detectable toxicity in mice [8]. Therefore, as a proof-of-principle we tested the effects of administering 40 µg of the peptide by intratracheal instillation on day-2 and day-6 on lung injury and fibrosis; more frequent intratracheal instillations in Bleomycin treated mice were not well tolerated.

Treatment of C/EBPβ^wt^ mice with the C/EBPβ peptide on day-2 and day-6 after receiving Bleomycin on day-0 also prevented progression of lung injury and fibrosis compared to control mice treated only with Bleomycin (**Figures 1** **,** **2** **,** **3** **and** **4**). These results also support the role of phosphorylated C/EBPβ-Thr217 in lung injury and fibrosis. A more comprehensive pharmacokinetic and pharmacodynamics analysis would be required to fully understand the effects of the C/EBPβ peptide on lung injury and fibrosis. This approach will necessitate the development of other forms of administering the peptide to animals with lung injury.

It would be important to determine whether RSK and phosphorylation of C/EBPβ are also critical in animal models that reflect other causes of human lung fibrosis, such as radiation-induced pneumonitis and fibrosis [18]. Any one of these studies will require as extensive an analysis as that performed with the Bleomycin model of lung fibrosis.

We found that mice expressing the C/EBPβ-Ala217 transgene were refractory to the induction of LMF activation by Bleomycin treatment. After Bleomycin administration, C/EBPβ was phosphorylated on Thr217 in LMF of C/EBPβ^wt^ mice, but not in LMF of C/EBPβ-Ala217 mice (**Figures 5** **and** **6**). Moreover, Bleomycin treatment induced the apoptotic cascade in LMF in the lungs of C/EBPβ-Ala217 mice, but not C/EBPβ^wt^ mice, as determined by the presence of active caspase 8 and RIP (**Figures 6** **and S4**). We have shown that C/EBPβ-Ala217 peptides directly activate procaspase 8 at picomolar concentrations in cell-free systems [10], explaining perhaps the activation of caspase 8 in the lungs of C/EBPβ-Ala217 mice 21 days after the Bleomycin exposure.

Also we found that inhibition of the MAPK, ERK1/2, RSK signaling (with ERK1/2 inhibitor or a dominant negative C/EBPβ peptide acting immediately upstream of C/EBPβ phosphorylation) in cultured, activated human LMF induces the apoptotic pathway. The apoptotic induction was identified by increased caspase 8 activation and annexin-V binding to phosphatidylserine in plasma membranes, an early indicator of apoptosis [10] (**Figure 8A, 8B and 8E**). ERK1/2 or RSK inhibitors also blocked expression of fibrogenic indicators α-SMA and TGF-β1 in cultured, activated human LMF (**Figure 8C and 8D**). Collectively, these data in cultured, activated human LMF supports the hypothesis that the MAPK, ERK1/2, RSK signaling upstream of C/EBPβ phosphorylation is important for lung fibrogenesis. These findings would be enhanced by demonstrating that ex vivo activation of LMF from both C/EBPβ-Ala217 transgenic mice and from control C/EBPβ^wt^ mice (after treatment in culture with ERK1/2, or dominant negative C/EBPβ-Ala217 peptides) also induces the apoptotic cascade, as we reported with liver myofibroblasts [10]. However, isolation and characterization of LMF from the different animal models will require extensive additional methodological validation and investigation.

C/EBPβ protein expression was induced by Bleomycin treatment in the lungs of C/EBPβ^wt^ mice but not in the lung of C/EBPβ-Ala217 transgenic mice (**Figure 6**), suggesting that lung C/EBPβ protein expression is linked to lung injury/fibrogenesis and/or requires RSK activity, which is suppressed by the C/EBPβ-Ala217 transgene (**Figure 6**). It remains to be determined what are the mechanisms modulating C/EBPβ expression under these experimental conditions. We and others have reported that the expression of C/EBPβ is regulated both at the transcriptional and post-transcriptional levels [41]–[43]. For example, the size of the C/EBPβ mRNA pool is rapidly increased by LPS and IL-1 indicating a transcriptional activation of *c/ebpβ* or increased C/EBPβ mRNA stability [42]. Given the inflammatory response elicited in Bleomycin-induced lung injury it is plausible that this mechanism is operative in these animals. We reported that mRNA levels in lung result in a rate of synthesis of C/EBPβ protein ∼100-fold less than that of the liver [41], suggesting a less efficient lung-specific translational regulation of C/EBPβ [44] through repression of translation from the C/EBPβ mRNA in the lung [43]. Perhaps, de-repression of C/EBPβ mRNA translation occurs in Bleomycin-induced lung injury.

C/EBPβΔ154 (also known as LIP), an alternative, shorter translation product initiated at the third AUG codon of the C/EBPβ mRNA [35], was expressed in the lungs of C/EBPβ^wt^ mice after Bleomycin treatment (**Figure 6A**). The ratio of C/EBPβ: C/EBPβΔ154 may affect cell proliferation and differentiation, since it changes during development [35] and in LPS-mediated acute-phase response [43]. It is unknown whether this lung C/EBPβ mRNA translational regulation also occurs in human lung injury. However, in preliminary studies we found that C/EBPβΔ154 was also the main translation product in the lungs of patients with IPF (unpublished observations). In primary activated human LMF, C/EBPβ full length was also expressed as a lesser translation product initiated at the second AUG codon of the C/EBPβ mRNA [35] (**Figure 8A**). The mechanisms responsible for and the significance of expressing C/EBPβΔ154 remain to be elucidated.

C/EBPβ-Ala217 was co-localized with caspase 8 and induced the apoptotic cascade in activated, mouse and human LMF. The combined results suggest, but do not prove, a functional link between inactive RSK and the active caspase 8 complex II. Further, we reported the association between inactive RSK and active caspase 8 with C/EBPβ-Ala217 or unphosphorylated C/EBPβ, suggesting also a physical link [10]. However, identification of a putative structural RSK/caspase 8 complex would require crystallographic analysis.

C/EBPβ-Ala217 mice have a marked decrease in lung injury and inflammation on day-13 after Bleomycin treatment when compared to control C/EBPβ^wt^ mice (**Figures 3** **and** **4**). These findings are supported by our previous report on the inhibitory effect of C/EBPβ-Ala217 on macrophage activation in culture [17]. C/EBPβ appears to be a critical signaling molecule for macrophages since its expression is dramatically increased during differentiation of these cells, and it is induced by macrophage modulators (LPS, IL-1, G-CSF, TGFβ, vitamin D, retinoic acid) [45],[46]. We have proposed a role for phosphorylated C/EBPβ-Thr217 on the modulation of macrophage function and survival following their activation by tissue injury [17]. This hypothesis is congruent with the fact that expression of the dominant positive phosphorylation mimic C/EBPβ-Glu217 [15] enhances function and survival of macrophages [17], while C/EBPβ^ko^ macrophages display defective bacterial killing and tumor cytotoxicity [16]. In addition, the Bleomycin-induced protein expression of inflammatory cytokines (IL-1-α, IL-2, IL-3, IL-4, IL-5, IL-6, IL-10, TNF-α, IFN-γ and GM-CSF) was ameliorated in the lungs of C/EBPβ-Ala217 transgenic mice as well as in the lungs of C/EBPβ^wt^ mice receiving the peptide. Most likely, these findings reflect the role of C/EBPβ-Thr217 phosphorylation in enhancing transcriptional activity, without increasing DNA-binding affinity, from cytokine promoters as we reported for the corresponding rat C/EBPβ-Ser105 phosphorylation [47].

Analysis of lung macrophage activation into pro-inflammatory M1 and/or anti-inflammatory M2 phenotypes [48] in animal models and in human lung injury will be required to elucidate the mechanisms responsible for lung injury and inflammation mediated by mouse C/EBPβ-Thr217 or human C/EBPβ-Thr266. Additional sequential studies after Bleomycin exposure and in other animal models would be needed to discriminate whether decreased lung inflammation and injury contributes significantly to the inhibition of lung fibrosis in C/EBPβ-Ala217 mice. A corollary of our study is that mice expressing the dominant positive C/EBPβ-Glu217 transgene would be more susceptible to myofibroblast cell activation and lung fibrosis induced by lung injury and inflammation.

Although the specific association between C/EBPβ-PhosphoThr217 and inactive procaspase 8 is linked to the inhibition of active caspase 8 [8],[10] the precise molecular mechanisms by which phosphorylated C/EBPβ prevents the lung injury-induced apoptotic cascade in LMF have not been characterized yet. Phosphorylated C/EBPβ could inhibit pro-apoptotic proteins, such as p53 [49] or the activation of survival proteins, such as MnSOD [50], or FLIP [51]. Alternatively, granzyme B rather than caspase 8 could be the major target of phosphorylated C/EBPβ-Thr217, as we suggested previously for liver myofibroblast apoptosis/survival [8],[10]. However, these potential mechanisms will have to be assessed experimentally in models of lung fibrosis.

These results suggest that phosphorylation of C/EBPβ-Thr266 (RSK phosphoacceptor) may be an important signaling pathway in human lung fibrosis. Lungs from patients afflicted with severe IPF, displayed a high level expression of both active, phosphorylated RSK and phosphorylated C/EBPβ-Thr266 co-localized in activated LMF within the fibrous tissue, compared with samples from control patients (**Figures 9** **and S5**). Thus, our findings in lung biopsies from patients with lung fibrosis are consistent with the hypothesis we developed in cellular and animal models implicating RSK activation and its phosphorylation of C/EBPβ in activated LMF in the development of human lung fibrosis.

Our data indicate that the RSK-C/EBPβ phosphorylation pathway may be critical for the progression of lung injury to lung fibrosis in the Bleomycin animal model and that it is activated in human lung fibrosis.

### Clinical Implications

This study suggest that blocking the RSK-C/EBPβ phosphorylation pathway inhibits fibrogenesis directly, by inhibiting LMF activation and indirectly, by reducing lung injury. There is no available effective treatment for lung fibrosis.

It is premature to assess whether this signaling pathway could be a potential target in the prevention and treatment of lung injury and fibrosis. Both lack of progression of lung fibrosis, as we documented in our study, as well as regression of lung fibrosis (a necessary aim of future investigations) in spite of continued lung injury are considered important clinical aims for patients with chronic lung disease and lung fibrosis [1],[5]–[7].

Finally, blocking the progression of lung fibrosis may decrease the need for lung transplantation since IPF is a main indication for lung transplants worldwide [2].

## Materials and Methods

### Ethics Statement

The human protocol was approved by the University of California, San Diego Human Protection Program project # 100380X, Project ID 1155365, IRB Protocol: 10-0380. For this research involving human participants, informed consent was exempted since the data were analyzed anonymously. All clinical investigation has been conducted according to the principles expressed in the Declaration of Helsinki. The animal studies were carried out in strict accordance with the recommendations in the Guide for the Care and Use of Laboratory Animals of the National Institutes of Health. The protocol was approved by the Committee on the Ethics of Animal Experiments of the San Diego VA Health Care System IACUC Protocol: 08-046.

### Animal Procedures

This study was carried out in strict accordance with the recommendations in the Guide for the Care and Use of Laboratory Animals of the National Institutes of Health. The protocol was approved by the Committee on the Ethics of Animal Experiments of the San Diego VA Health Care System. All tracheal instillations were performed under anesthesia, and all efforts were made to minimize suffering. The mice were given an IP injection of rodent anesthesia (0.4 ml/100 g of 50 mg/kg ketamine; 5 mg/kg xylazine; and 1 mg/kg acepromazine). We administered Bleomycin (2 U/kg in 50 µl saline by intratracheal instillation) or saline (50 µl) once to C/EBPβ^wt^ and C/EBPβAla217 mice [8] (23–27 g). In other experiments, C/EBPβ^wt^ mice (25 g) each received Bleomycin (2 U/kg by intratracheal instillation) once but also received the cell permeant C/EBPβ (Ac- K-Ala217-V-D-CHO) peptide (American Peptide; Sunnyvale, CA) [10] (40 µg by intratracheal instillation on day-2 and day-6). Animals were sacrificed on day 13 or on day-21 after the Bleomycin treatment.

### Construction of C/EBPβ-Ala217 mice

Transgenic mice expressing C/EBPβ-Ala217, a dominant negative, nonphosphorylatable mutation of the C/EBPβ-Thr217 phosphoacceptor, were generated as described previously [10] and back-crossed to the parental wild-type inbreed FVB mice for >10 generations. The mouse C/EBPβ cDNA was amplified by PCR to mutate Thr217. The primers used to mutate Thr217 to Ala217 were S 5′-GCC AAG GCC AAG AAG GCG GTG GAC AAG CTG AGC -3′ and AS 5′-GCT CAG CTT GTC CAC CGC CTT CTT GGC CTT GGC -3′. C/EBPβ was removed from pEVRF0 with Apa I and Nhe I and cloned into pHM6 vector (Boehringer-Mannheim, cat. # 1814664) in order to add an RSV promoter upstream to the C/EBPβ start site. C/EBPβ was removed from this new construct with Bsa I and Bsp. The 982 bp insert was cloned into mammalian vector pOP13CAT (Stratagene) from which the CAT portion had been previously removed. DNA for pronuclear injection was purified over a CsCl gradient and digested with Ssp I and EclHK I. The 3.6 Kb fragment was separated on gel electrophoresis on a 0.8% gel with no Ethidium Bromide. The appropriate band was removed and electro eluted from the gel using the Elutrap apparatus from Schleicher & Schuell. The eluted DNA was purified with a Qiagen-20 column, precipitated with isopropanol and dissolved in 7.5 mM TRIS pH 7.4, 0.15 mM EDTA. All solutions were prepared with tissue grade water, endotoxin tested (Gibco). The DNA was dialyzed and injected into fertilized ova at a concentration of 1.8 µg/ml (Transgenic Mouse Core Facilities, University of California, San Diego). The presence of the *rsv* gene was used to identify these transgenic mice by PCR, and 3 positive mice resulted. The primer sequences for the RSV PCR were custom designed (RSV.2271 TAGGGTGTGTTTAGGCGAAA sense and RSV.2510 TCTGTTGCCTTCCTAATAAG antisense). The PCR reagents were all from Quiagen. Transgene-bearing founder mice were mated with FVB mice. All founder mice produced viable offspring.

### Cell cultures

Primary human lung fibroblasts isolated from normal human lung parenchyma (Cell Applications; San Diego, CA) were cryopreserved at first passage and can be cultured and propagated at least 12 population doublings. Cells were freshly activated by culturing on a collagen type 1 matrix as we described [52]. Activated LMF were untreated or treated for up to 24 hr with the MAPK ERK1/2 inhibitor (10 or 20 µM) (Calbiochem 328006) or treated for up to 24 hr with the Ac- K-Ala217-V-D-CHO peptide (200 µM) [10] (American Peptide Co) and prepared for microscopy or cell lysates were immunoprecipitated with specific antibodies.

### Microscopy

Fluorescent labels were observed using antibodies against C/EBPβ, RSKPhosphoSer380, α-SMA (Abcam), SFPC (Abcam), CD68 (Genway) , CD45 (Abcam), RIP (Santa Cruz Biotechnology, Santa Cruz, California), or C/EBPβ-PhosphoThr217 [10] in a laser confocal scanning microscope [10],[15],[34]. Fluorochromes utilized were Alexa 488 and Alexa 594. At least 100 cells were analyzed per experimental point [53]. We used TO-PRO-3 (Molecular Probes, Eugene, Oregon) to analyze nuclear morphology. The number of annexin-V (+) LMF was determined by in vivo microscopy [10]. These values are reported as a percentage of annexin-V (+) LMF. The degree of lung injury and fibrosis was determined by using Hematoxylin/Eosin , Mallory's trichrome and Sirius red (under a polarized light) histochemistry [26],[54]. The inter-observer agreement was >85%.

The program that we used for immunohistochemistry quantitation was MetaMorph Offline, Universal Imaging Product version 6.1. The MetaMorph Offline software package is an accepted standard used to measure, analyze, and display data acquired from a confocal system. We used the MetaMorph Offline program to graph fluorescent intensities (integrated intensity; summed over all of the pixels in the region) in a defined area from the acquired confocal images. These data were exported directly to Excel (Microsoft) for the calculations. At least five random fields (×200) were analyzed per experimental point [28].

### Lung Fibrosis

We determined the degree of lung fibrosis in coded samples. We evaluated lung fibrogenesis employing the following methods as previously described [8]: i) trichrome stain; ii) Sirius red stain; iii) quantitative Sirius red collagen-binding assay; iv) quantitative collagen type 1 assay; v) quantitative hydroxyproline assay; vi) protein expression of α-smooth muscle actin (α-SMA); and vii) protein expression of transforming growth factor (TGF)-β1.

### Lung Inflammatory Cytokines

A multiplex protein cytokine detection system (Quansys Biosciences, Logan, UT) was utilized in lung tissue lysates according to the manufacturer's protocol to determine IL-1-α, IL-2, IL-3, IL-4, IL-5, IL-6, IL-10, TNF-α, IFN-γ and GM-CSF. Values calculated from individual pixels using the Q-View Imager system.

### Immunoprecipitation and Immunoblots

Pre-cleared lung or LMF lysates were incubated for 2 h with purified C/EBPβ, C/EBPβ-Phospho-Thr217, RSK, caspase 8 (Santa Cruz Biotechnology) , α-SMA, TGFβ or β-actin (Abcam) antibodies followed by the addition of A/G+ agarose (Santa Cruz Biotechnology) for 12 h. The immunoprecipitation reactions each contained 500 µg of total protein and 2 µg antibody (or purified IgG pre-immune serum as negative control). Immunoprecipitates were washed 3 times in 500 ml cell lysis buffer [41] and resolved by SDS-PAGE, and detected by western blot [47],[55], following the Odyssey Western Infrared Detection Method and Odyssey Visualization protocol using purified antibodies against C/EBPβ (C-19; aa 258–276), RSK, α-SMA, TGFβ, β-actin, procaspase 8 (PharMingen) and C/EBPβ-PhosphoThr217(h266) [10].

### Human Lungs

We obtained anonymous, de-identified lung samples from 2 patients with IPF and severe lung fibrosis (65-year old female and 61-year old male) and from 2 matched control subjects without lung disease (65-year old female and 60-year old male) (Arseland and ProSci).

### Statistical Analysis

Results are expressed as mean (± SD). The Student-*t* and the Wilcoxon U tests were used to evaluate the differences of the means between groups for parametric and non-parametric populations, respectively, with a *P* value of <0.05 as significant.

## Supporting Information

Figure S1 **RSK inhibition blocks lung injury induced by Bleomycin.** **A.** Mice were from the experiment described in (Fig. 1). Analysis was performed on day-13. Representative hematoxylin and eosin stain for lung injury. C/EBPβ^wt^ mice treated with Bleomycin developed severe lung injury. The C/EBPβ-Ala217 mice treated with Bleomycin had only minimal or moderate lung injury. Treatment of C/EBPβ^wt^ mice with the C/EBPβ peptide (on day-2 and day-6) after Bleomycin treatment decreased the development of lung injury. The bars represent 50 µm.
(TIF)

Figure S2 **Mice expressing the C/EBPβ-Ala217 transgene are resistant to Bleomycin-induced lung inflammation.** Mice were treated with Bleomycin and confocal microscopy was performed as described in Methods. **A.** Activated monocytes/macrophages, identified by confocal microscopy for CD68^+^ (green), were increased at day-13 in the lungs of C/EBPβ ^wt^ mice treated with Bleomycin, but not in the lungs of C/EBPβ-Ala217 mice after treatment with Bleomycin or in the lungs of C/EBPβ ^wt^ treated with Bleomycin and the C/EBPβ peptide. Nuclei are identified with TO-PRO-3 (blue). Only background staining was observed when omitting the first antibody. Microscopy shown is representative of each group. The bars represent 50 µm.
(TIF)

Figure S3 **Induction CD45^**+**^ cells in the lungs of Bleomycin-treated C/EBPβ ^wt^ mice.** Mice were treated with Bleomycin as described in (Fig. 1) and confocal microscopy was performed as described in Methods. CD45^+^ (red) was induced and co-localized at day-13 in the lungs of C/EBPβ^wt^ treated with Bleomycin, but not in C/EBPβ-Ala217 mice treated with Bleomycin or in the lungs of C/EBPβ^wt^ treated with Bleomycin and the C/EBPβ peptide. Nuclei are identified with TO-PRO-3 (blue). Only background staining was observed when omitting the first antibody. The bar represents 50 µm.
(TIF)

Figure S4 **RIP is induced in the lungs of mice expressing the C/EBPβ-Ala217 transgene.** Mice were treated with Bleomycin as described in (Fig. 1) and confocal microscopy was performed as described in Methods. RIP (red) was induced at day-21 in the lungs of C/EBPβ-Ala217 mice treated with Bleomycin and in the lungs of C/EBPβ ^wt^ treated with Bleomycin and the C/EBPβ peptide, but not in C/EBPβ ^wt^ treated with Bleomycin. Nuclei are identified with TO-PRO-3 (blue). Only background staining was observed when omitting the first antibody. The bar represents 50 µm.
(TIF)

Figure S5 **Induction and co-localization of active RSK and C/EBPβ-PhosphoThr266 in human lung fibrosis.** Representative confocal microscopy of 2 IPF patients with severe lung fibrosis and 2 matched control subjects. Activated LMF, identified by confocal microscopy for their morphology and α-SMA expression (as in Fig. 8) , displayed activated RSK-PhosphoSer380 (red) and C/EBPβ-PhosphoThr266 (green) in lungs of IPF patients with severe lung fibrosis (lower panel) but not in the lungs of control subjects (upper panel). Co-localization of RSK-PhosphoSer380 and C/EBPβ-PhosphoThr266 is shown in yellow or white (merge). Nuclei are identified with TO-PRO-3 (blue). Only background staining was observed when omitting the first antibody.
(TIF)
