# Supplementary figures and images for: C/EBPβ-Thr217 Phosphorylation Signaling Contributes to the Development of Lung Injury and Fibrosis in Mice
Source: PLoS One. 2011 Oct 5;6(10):e25497. doi: 10.1371/journal.pone.0025497 (PMC3187778; doi:10.1371/journal.pone.0025497)

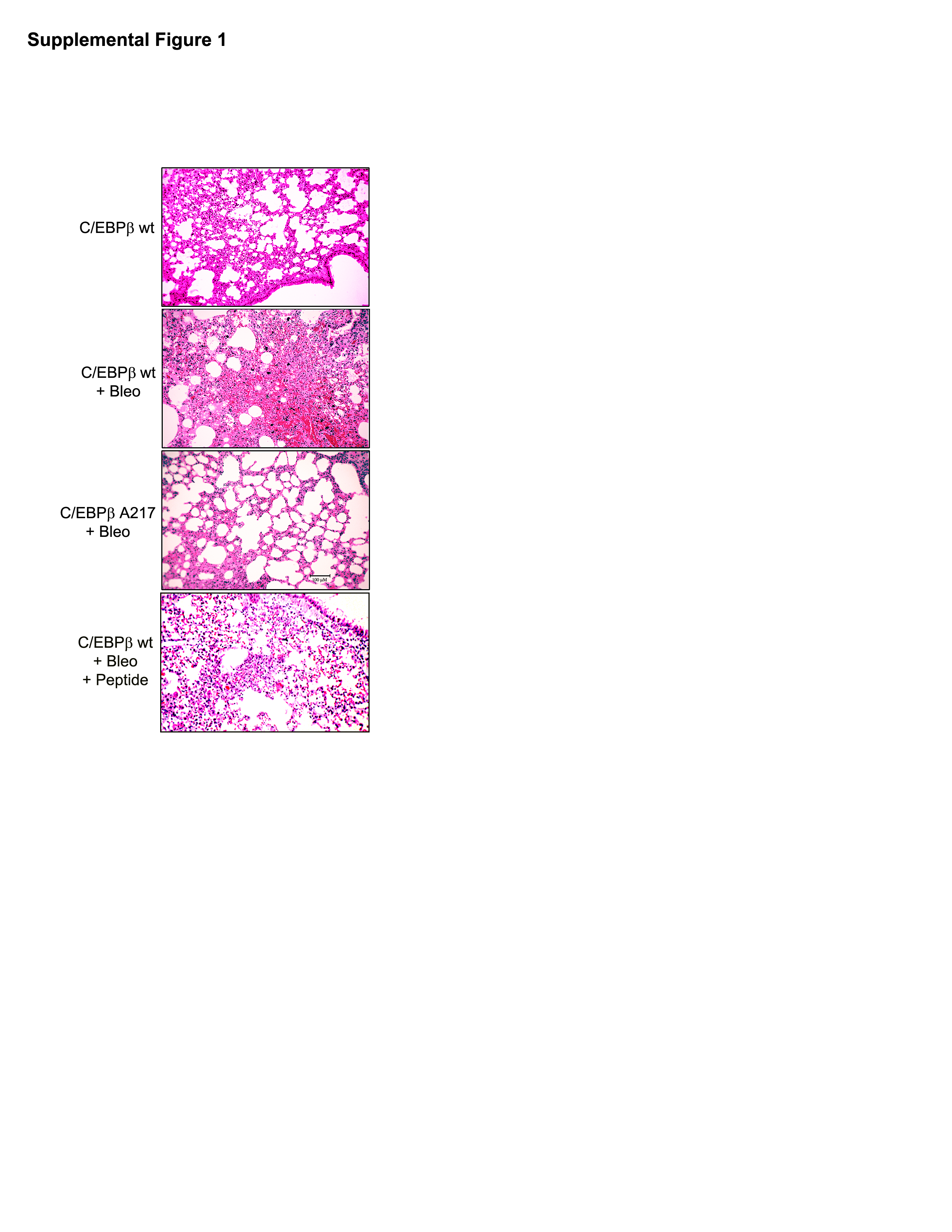

Supplement: Figure S1 — RSK inhibition blocks lung injury induced by Bleomycin. A. Mice were from the experiment described in (Fig. 1). Analysis was performed on day-13. Representative hematoxylin and eosin stain for lung injury. C/EBPβwt mice treated with Bleomycin developed severe lung injury. The C/EBPβ-Ala217 mice treated with Bleomycin had only minimal or moderate lung injury. Treatment of C/EBPβwt mice with the C/EBPβ peptide (on day-2 and day-6) after Bleomycin treatment decreased the development of lung injury. The bars represent 50 µm. (TIF) [file pone.0025497.s001.tif]

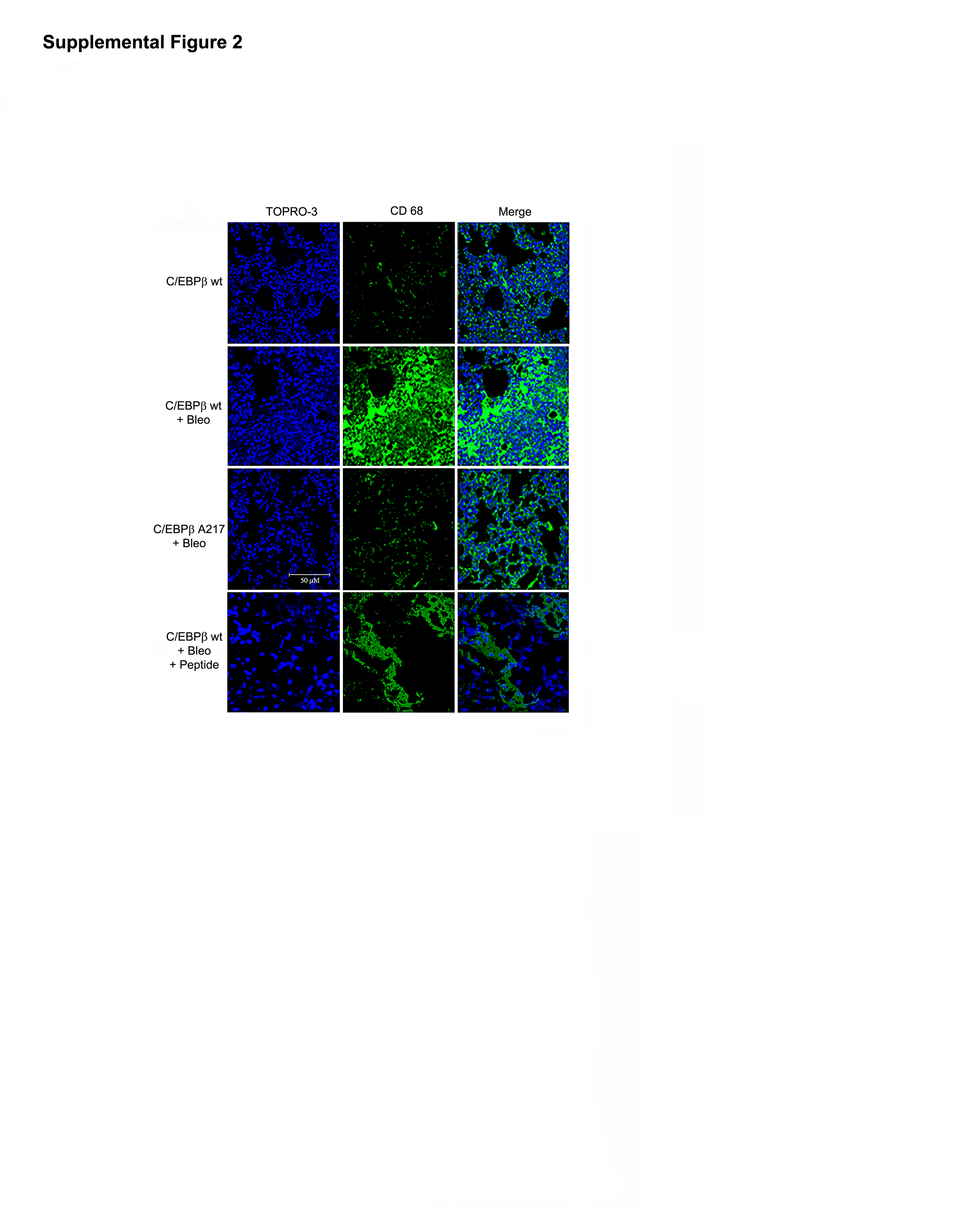

Supplement: Figure S2 — Mice expressing the C/EBPβ-Ala217 transgene are resistant to Bleomycin-induced lung inflammation. Mice were treated with Bleomycin and confocal microscopy was performed as described in Methods. A. Activated monocytes/macrophages, identified by confocal microscopy for CD68+ (green), were increased at day-13 in the lungs of C/EBPβ wt mice treated with Bleomycin, but not in the lungs of C/EBPβ-Ala217 mice after treatment with Bleomycin or in the lungs of C/EBPβ wt treated with Bleomycin and the C/EBPβ peptide. Nuclei are identified with TO-PRO-3 (blue). Only background staining was observed when omitting the first antibody. Microscopy shown is representative of each group. The bars represent 50 µm. (TIF) [file pone.0025497.s002.tif]

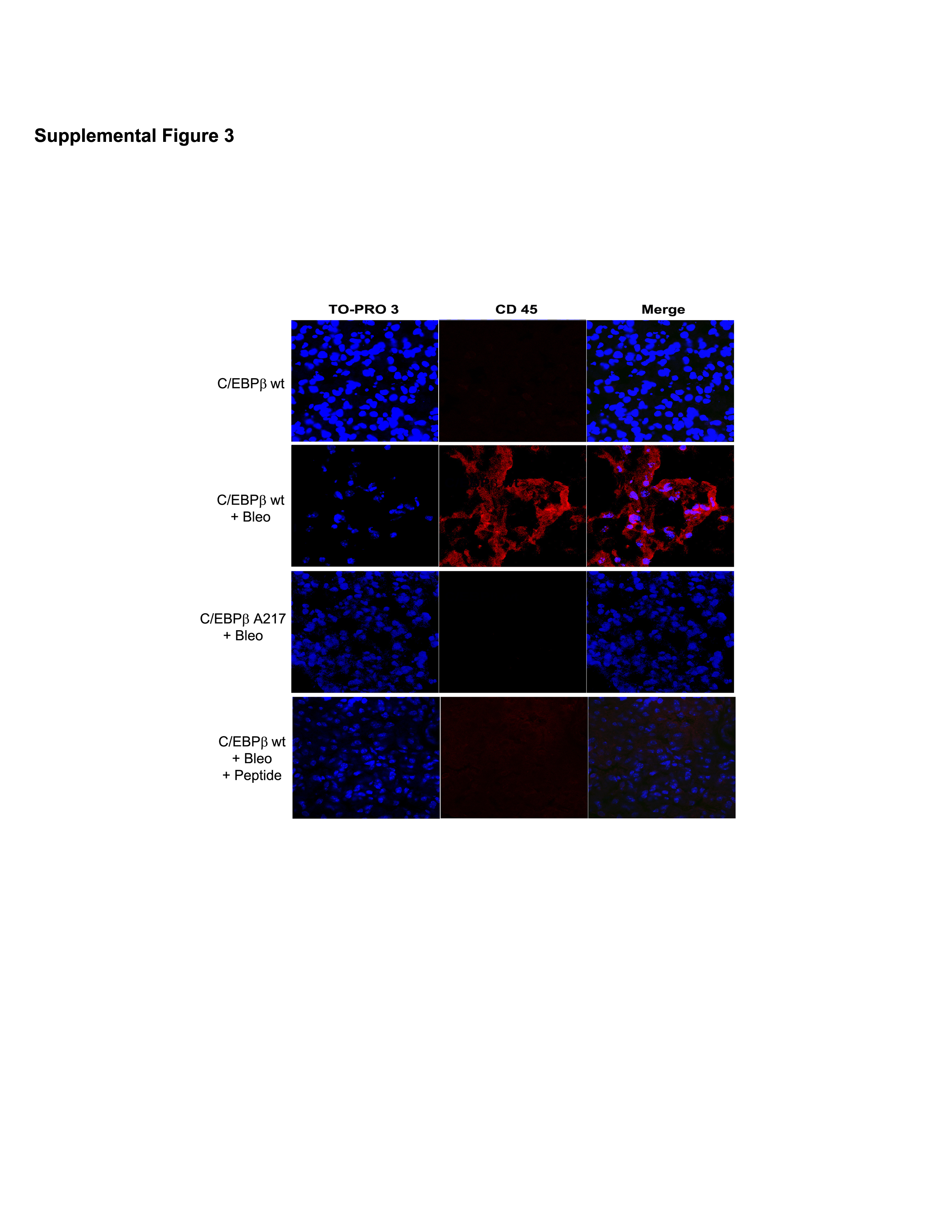

Supplement: Figure S3 — Induction CD45+ cells in the lungs of Bleomycin-treated C/EBPβ wt mice. Mice were treated with Bleomycin as described in (Fig. 1) and confocal microscopy was performed as described in Methods. CD45+ (red) was induced and co-localized at day-13 in the lungs of C/EBPβwt treated with Bleomycin, but not in C/EBPβ-Ala217 mice treated with Bleomycin or in the lungs of C/EBPβwt treated with Bleomycin and the C/EBPβ peptide. Nuclei are identified with TO-PRO-3 (blue). Only background staining was observed when omitting the first antibody. The bar represents 50 µm. (TIF) [file pone.0025497.s003.tif]

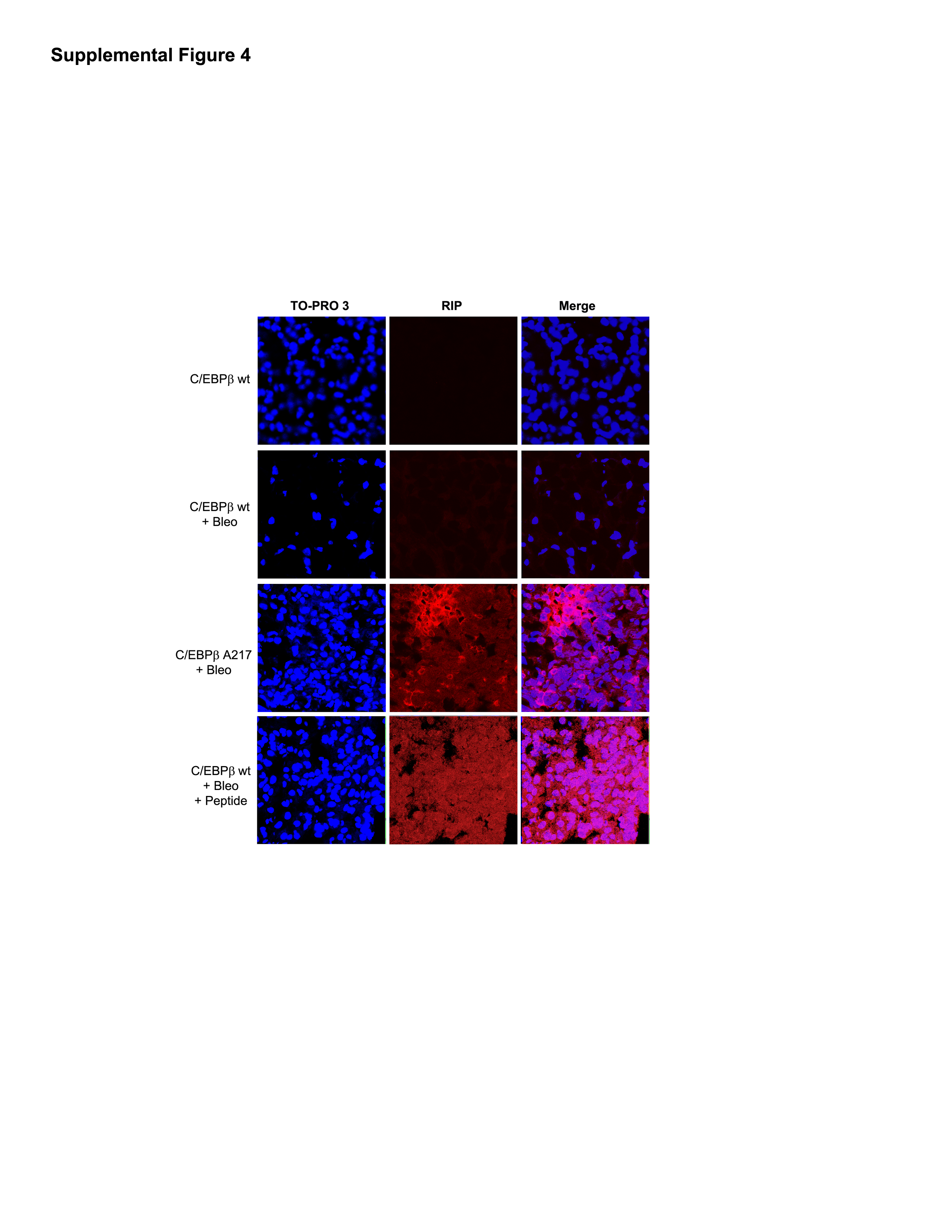

Supplement: Figure S4 — RIP is induced in the lungs of mice expressing the C/EBPβ-Ala217 transgene. Mice were treated with Bleomycin as described in (Fig. 1) and confocal microscopy was performed as described in Methods. RIP (red) was induced at day-21 in the lungs of C/EBPβ-Ala217 mice treated with Bleomycin and in the lungs of C/EBPβ wt treated with Bleomycin and the C/EBPβ peptide, but not in C/EBPβ wt treated with Bleomycin. Nuclei are identified with TO-PRO-3 (blue). Only background staining was observed when omitting the first antibody. The bar represents 50 µm. (TIF) [file pone.0025497.s004.tif]

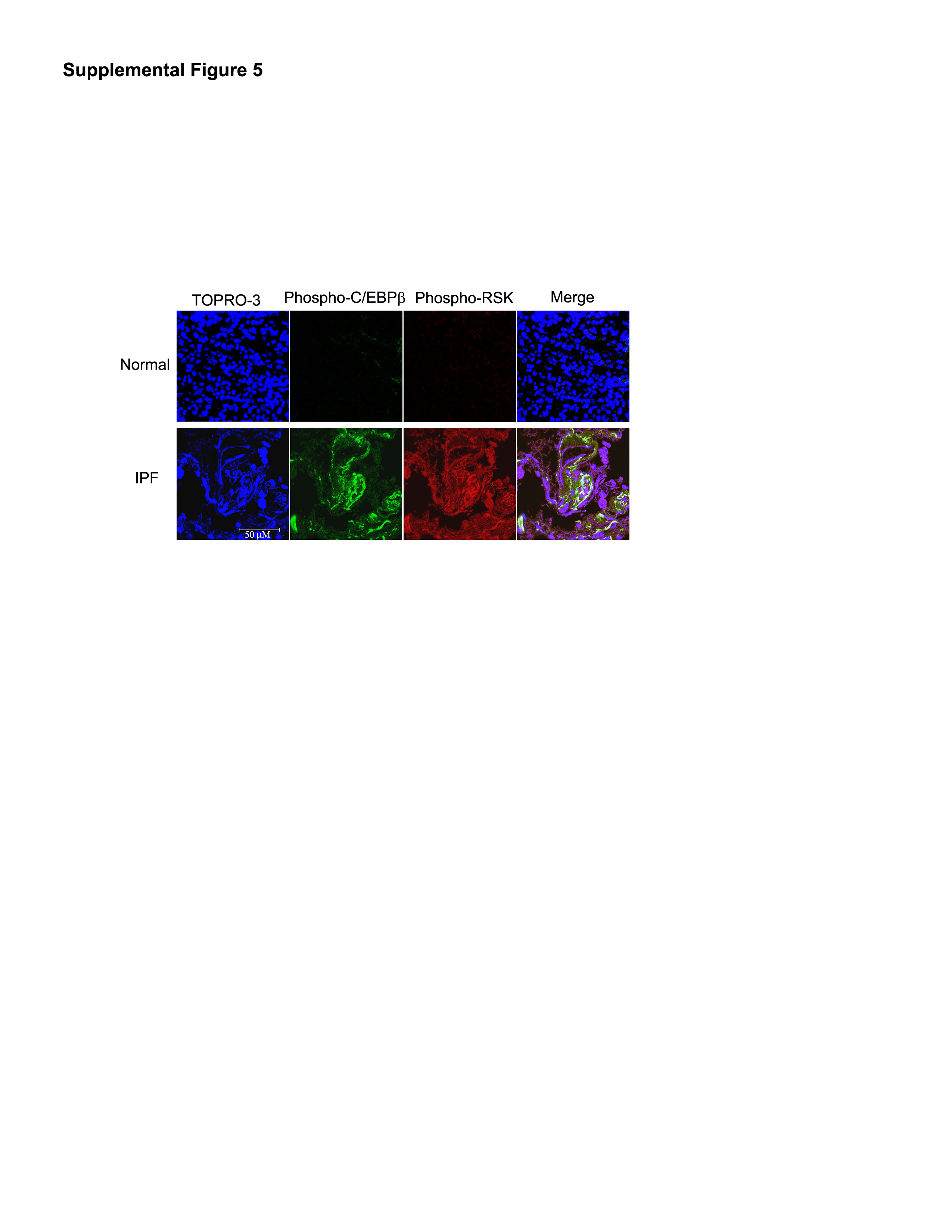

Supplement: Figure S5 — Induction and co-localization of active RSK and C/EBPβ-PhosphoThr266 in human lung fibrosis. Representative confocal microscopy of 2 IPF patients with severe lung fibrosis and 2 matched control subjects. Activated LMF, identified by confocal microscopy for their morphology and α-SMA expression (as in Fig. 8) , displayed activated RSK-PhosphoSer380 (red) and C/EBPβ-PhosphoThr266 (green) in lungs of IPF patients with severe lung fibrosis (lower panel) but not in the lungs of control subjects (upper panel). Co-localization of RSK-PhosphoSer380 and C/EBPβ-PhosphoThr266 is shown in yellow or white (merge). Nuclei are identified with TO-PRO-3 (blue). Only background staining was observed when omitting the first antibody. (TIF) [file pone.0025497.s005.tif]
